# Locomotion engages context-dependent motor strategies for head stabilization in primates

**DOI:** 10.1038/s42003-026-09512-2

**Published:** 2026-01-12

**Authors:** Rui-Han Wei, Oliver R. Stanley, Adam S. Charles, Kathleen E. Cullen

**Affiliations:** 1 Department of Biomedical Engineering, Johns Hopkins University, Baltimore, MD USA; 2 Mathematical Institute for Data Science, Kavli Neuroscience Discovery Institute & Center for Imaging Science, John Hopkins University, Baltimore, MD USA; 3 Kavli Neuroscience Discovery Institute, Johns Hopkins University, Baltimore, MD USA; 4 Department of Otolaryngology-Head and Neck Surgery, Johns Hopkins University School of Medicine, Baltimore, MD USA; 5 Department of Neuroscience, Johns Hopkins University School of Medicine, Baltimore, MD USA

**Keywords:** Motor control, Biomechanics

## Abstract

Flexible motor control is essential for navigating complex, unpredictable environments. Although movement execution is often associated with stereotyped patterns of neural and muscular activation, the degree to which these patterns are conserved versus flexibly reorganized to meet task demands across diverse contextual changes has not been well characterized. Here we recorded head and body kinematics alongside muscle activity in rhesus monkeys during head stabilization—crucial for maintaining gaze and balance—while walking on a treadmill at various speeds, and during overground locomotion in the presence or absence of enhanced autonomic arousal. Dimensionality reduction analyses revealed a flexible control strategy during treadmill walking: a stable activation structure that scaled with speed. In contrast, overground walking evoked heightened muscle engagement and more substantial changes in organization. This pattern largely persisted even during elevated arousal, with larger pupil size linked to stronger but structurally preserved muscle recruitment. Together these findings demonstrate that the brain dynamically adapts motor coordination to context even for automatic behaviors, underscoring the need to examine control strategies in a wide range of conditions.

## Abstract (web summary)

Head stabilization arises from population geometry of neck muscle activity that is conserved and smoothly scaled across walking speeds, but reorganized during more natural overground locomotion, revealing flexible, context-dependent motor control.

## Introduction

Flexible motor control is essential for navigating and interacting with complex, unpredictable environments, yet how the brain achieves such adaptability for foundational motor tasks such as balance and locomotion remains poorly understood. One such task, head stabilization during locomotion, is particularly important because it anchors visual and vestibular processing^1,2^ and thereby underlies posture and navigation across contexts. Previous work on head stabilization during locomotion has primarily characterized the kinematics of head motion, showing consistent gait-phase-locked patterns across species (cats^3,4^; horses^5,6^; monkeys^7–9^; humans^10–16^). However, few studies have examined the underlying recruitment of neck muscles. These reports have focused largely on activation timing, revealing gait-cycle-dependent muscle engagement in both quadrupeds^6,17,18^ and humans^19,20^. Since achieving stable head posture during locomotion involves coordination among neck muscles, a deeper understanding of head stabilization requires analysis of activity across multiple muscles simultaneously.

Previous studies of limb and trunk muscles have shown that complex, multi-muscle activation patterns can be captured by low-dimensional population structures^21–28^. Across changes in posture, speed, and locomotor mode, these motor synergies retain a conserved organization with shifts in exact timing or amplitude, suggesting that the nervous system flexibly coordinates established patterns rather than generating new activation sequences for each condition. For example, in human locomotion, the fundamental EMG components change little with treadmill speed^22^, running exhibits a comparable modular structure on the treadmill and overground^28^, and treadmill walking produces more temporally constrained activation than overground walking^24^. Likewise, studies in nonhuman primates have revealed context-dependent adjustments in muscle coordination across locomotor modes, for instance, between bipedal and quadrupedal^29,30^, or between bipedal and climbing or quadrupedal^31^, behaviors. Together, these findings point to a general principle of low-dimensional, reusable modules that enable efficient adaptation to biomechanical and sensory demands. However, such principles have been explored almost exclusively in limb and trunk muscles. Whether similar low-dimensional coordination governs head stabilization, and how this control flexibly reorganizes across contexts, remains unknown.

To address this question, we investigated neck muscle coordination supporting head stabilization during locomotion in nonhuman primates. Specifically, we quantified 1) head and body kinematics to quantify stabilization performance 2) bilateral neck muscle EMG and motor unit activity from the splenius capitis and sternocleidomastoid (SPL and SCM, respectively) to evaluate the single muscle responses, and 3) population-level activation patterns to identify motor control strategies across contexts. In addition to biomechanical factors, internal state—including perceived threat^32^, mood^33^, or arousal^34^—can modulate both kinematics and muscle recruitment. Accordingly, we recorded across a range of contexts: animals walked on an externally-paced treadmill at multiple speeds and during self-paced overground locomotion, both with and without the presence of a friendly conspecific (see ‘Autonomic arousal paradigm’, Methods). Following work in the neural motor control literature^35–40^, we defined motor control strategies within a latent space framework, as trajectories derived from recruitment sequences and relative activation weights captured by population-level structure across contexts. Here, “context” refers to a specific combination of mechanical demands, sensory inputs (optic flow, vestibular, and proprioceptive), and internal state, with the behavioral goal held constant.

Although head stabilization was maintained across all conditions, the underlying control strategies diverged. During treadmill walking, neck muscle activity followed a consistent, speed-scaled activation pattern, reflecting a stable coordination scheme tuned to biomechanical load. In contrast, overground locomotion evoked a more significantly altered organization of muscle activity, indicating that self-paced movement engages a modified control strategy. This organization remained largely preserved under heightened autonomic arousal: pupil-linked increases in neck muscle activity scaled response amplitude with minimal disruption of the underlying population geometry. Together, these findings reveal that motor coordination is not governed by a fixed, universal strategy. Instead, the brain dynamically reconfigures muscle population activity to meet contextual demands, providing a fundamental mechanism by which motor systems preserve stable behavior across conditions.

## Results

**Fig. 1:**
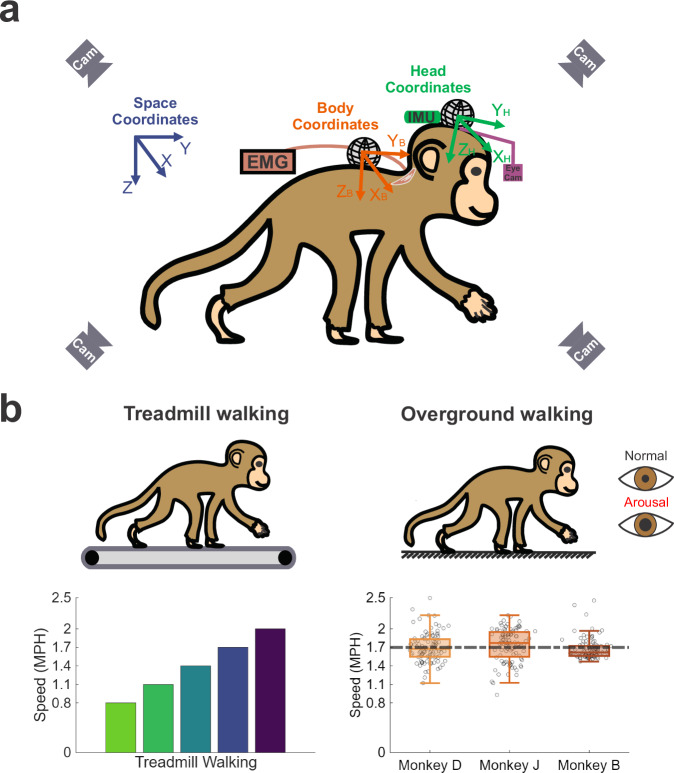
Schematic of the experimental setup. **a** Illustration of data collection (EMG and kinematics) and coordinate frames for head and body movements in space for a monkey. **b** Illustrations of treadmill walking (left) and overground walking (right) under normal and arousal conditions. Bottom plots illustrate all treadmill walking speeds (0.8, 1.1, 1.4, 1.7, and 2.0 MPH) and the average walking speed of each monkey during overground walking (1.70 ± 0.019, 1.76 ± 0.027 and 1.65 ± 0.017 MPH, for monkey D, monkey J and monkey B, respectively; *p* > 0.05; (*N* = 100 for each monkey), which matches one of the treadmill speeds used (1.7 MPH, green dashed line).

Our goal was to determine whether the brain’s motor control strategy for stabilizing the head during locomotion adapts across behavioral contexts. We simultaneously recorded 3D limb kinematics, 6D head and body motion, and motor unit activity from four neck muscles (two bilateral pairs) in nonhuman primates walking on a treadmill (externally paced) and overground (self-paced) (Fig. 1). These conditions allowed systematic examination of how speed, propulsion mode, and autonomic arousal influence head stabilization. We first assessed how head-in-space and head-on-body kinematics varied with treadmill speed, then compared treadmill and overground walking at matched speeds to determine the effect of locomotor control mode. To then determine how contextual differences arose at the level of motor output, we analyzed neck muscle activity both individually and as a population. Finally, to test the influence of internal state, we compared overground walking under baseline and heightened autonomic arousal. For kinematic analyses, we quantified head position, velocity, and acceleration using amplitude and total variation to assess head stabilization performance across contexts. Head-on-body motion was additionally evaluated to determine how compensatory movements contribute to stabilization. For EMG, we quantified recruitment amplitude at both the single- and multi-motor unit level, and derived low-dimensional latent representations of population activity to characterize activation structure. This framework allowed us to test whether motor control relies on a conserved coordination strategy or reorganizes flexibly across behavioral contexts.

### Head-in-space stabilization is enhanced during overground versus treadmill walking

**Fig. 2:**
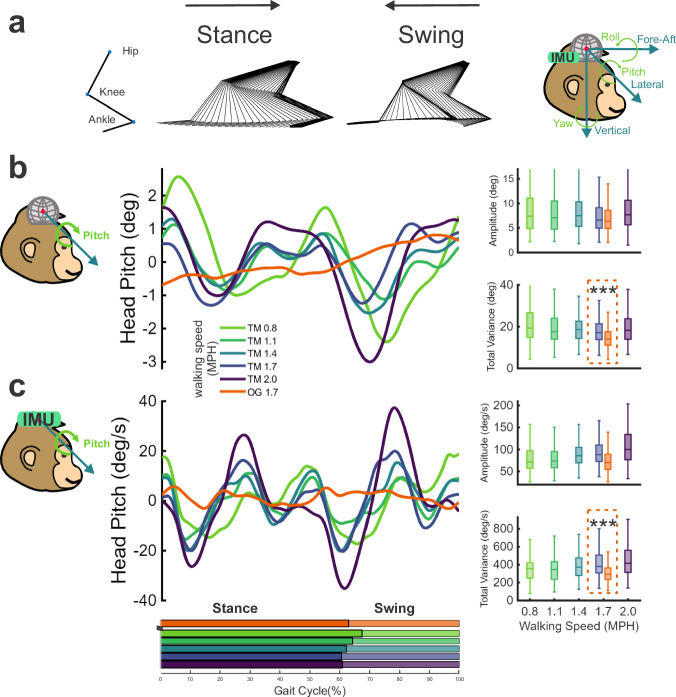
The head is well-stabilized in space across walking conditions. **a** Illustration of gait and head kinematic data. Stick diagrams at the top show successive hindlimb positions during the stance and swing phases, reconstructed from a representative gait cycle at 1.7 MPH on the treadmill. **b**, **c** Time course of gait-cycle averaged head-in-space pitch angular position (**b**) and angular velocity (**c**) in representative Monkey J. Horizontal bars below indicate the stance and swing phase for each condition. Right-side panels show the Amp. (top) and V_Total_ (bottom) for each condition from the data of all monkeys. The average amplitude of position was consistent across speeds except between the two highest (TM 2.0 > TM 1.7, *p* = 0.0156). Total variance also remained consistent, except that it was slightly higher at the lowest speed (TM 0.8 > TM 2.0, *p* = 0.0145). Velocity amplitude and V_Total_ increased at higher speeds except for TM 0.8 vs TM 1.1 (both amplitude and V_Total_) and TM 1.4 vs TM 1.7 (amplitude). Results are presented as boxplots, which show the median (center line), interquartile range (box), and whiskers extending to the nonoutlier (1.5 IQR) maximum and minimum across 300 strides (100 strides per monkey). Color: TM treadmill walking: 0.8 (light green), 1.1 (green), 1.4 (cyan), 1.7 (blue), and 2.0 (purple) MPH, OG overground walking (orange). ***p < 0.001; all *p* values are reported in Supplementary Tables 1, 2, 4, 5.

Locomotion in different contexts naturally imposes varied motor demands. To understand the impact of context on the underlying motor control strategy for stabilizing the head, we first investigated head kinematics during locomotion at several treadmill speeds, aligned to the gait cycle (onset of stance phase, Fig. 2a). We measured head stabilization in each axis via amplitude, defined as the peak-to-trough difference within a gait cycle, and total variation (*V*_Total_), defined as the sum of timepoint-by-timepoint differences within a gait cycle, with lower values indicating better head stabilization. Even in the least stable rotation axis (pitch), average amplitude was <10° (Fig. 2b, right panel) and was generally consistent across speeds (Table S1). *V*_Total_ showed a comparable pattern, with minimal variation across speeds (Table S2). Similarly, in the least stable translation axis (vertical, Fig. S1a), mean amplitude was not significantly different across most speeds (Table S1), while the mean total variation was generally lower at higher speeds (Tables S2). Both amplitude and total variation were even lower in other axes—lateral and fore-aft translation, roll and yaw rotation (Fig. S1b–e). Notably, roll and yaw position (Fig. S1d, e) showed almost no significant differences across speeds, indicating consistent rotational head stabilization performance regardless of mechanical demand. To visualize the full data distributions, we plotted estimated means and confidence intervals from our linear mixed-effects (LME) models, superimposed on the individual data points (Fig. S2). Results for each specific subject are presented separately in Fig. S3, with individual *r* and *p* values reported in Table S3 for within-subject linear regressions. Together, these analyses revealed that head position amplitude and total variation remained consistent, or even decreased, with increasing speed across most axes—indicating robust head stabilization despite changes in locomotor mechanics.

Higher walking speeds generate larger forces and accelerations, which in turn pose greater challenges to maintaining a stable head position. We therefore hypothesized that maintaining head stabilization at higher walking speeds would require greater modulation of head motion across the gait cycle, reflected in changes at the level of higher-order kinematics. To test this possibility, we examined angular head velocity and linear head acceleration. We found that both amplitude and V_Total_ increased at higher speeds for pitch angular velocity (Fig. 2c; Tables S4, S5) and vertical linear acceleration (Fig. S4a; Tables S4 and S5). Indeed, both measures increased as a function of treadmill speed across all other axes (Fig. S4b–e, right panels). Detailed data distributions are plotted in Fig. S5. Detailed per-animal results are presented in Fig. S6, with individual r and *p* values listed in Table S6 for within-subject linear regressions. These findings are consistent with our hypothesis that the greater mechanical demands imposed at higher walking speeds are compensated for by increased velocity and acceleration to maintain a stable head position. Consequently, the minimal changes in head displacement observed at faster speeds likely reflect a tradeoff between position and higher-order kinematic components.

Although treadmills provide experimental control and are more space efficient in a laboratory setting, they do not capture the full complexity of natural movement. Consistent with this limitation, prior human studies have reported significant kinematic differences between treadmill and overground locomotion^41–43^. Accordingly, to explore whether similar differences exist in head stabilization, we extended our analysis to head movement during overground walking. Monkeys were trained to walk along a linear track for a reward (see Methods). Their overground speed averaged ~1.7 MPH and did not differ significantly between individuals (Fig. 1b, right bottom panel, *p* > 0.05). When compared at this matched speed, head position stabilization across the gait cycle was better for overground walking than for treadmill walking (Figs. 2b and S1, compare orange and blue traces). In particular, amplitude was reduced in the vertical axis (Fig. S1a, top right panel; *p* = 8.13E-4, Table S1) and total variation was lower in pitch and vertical position (Figs. 2b and S1a; *p* = 6.73E-11 & 0.0039; Table S2). Amplitude increased for lateral and fore-aft position, which likely resulted from an increase in voluntary visual exploration during the overground paradigm (Fig. S1b, c, e). Similar increases would also be expected during overground walking on surfaces that heighten visual demands for precise foot placement^44^. Beyond these changes in head position patterns, overground walking consistently produced lower head-in-space rotational velocity and translational acceleration (Figs. 2c and S3; compare orange and blue traces). Specifically, the amplitude of rotational velocity and linear acceleration was lower in every axis except yaw (Table S4), and total variation was lower for every axis (Table S5). Individual animal results and accompanying statistics are presented in Fig. S6 and Table S6, respectively. This difference suggests that animals were more effectively able to maintain head position stabilization during self-paced walking, achieving comparable stabilization with reduced reliance on changes in higher-order dynamics.

### Head stabilization is achieved via robust head-on-body compensation across contexts

We next asked how differences in head stabilization, both within and across contexts, were achieved through head-on-body movement. Neck muscle activation stabilizes the head in space by generating forces that counteract body motion. To better understand this mechanism during locomotion, we compared head-on-body motion to body-in-space motion. Specifically, we quantified compensation in each axis using gain and phase: gains near 1 indicate head-on-body movement of similar magnitude to the body, while phases near 180° reflect movement in the opposite direction (see Methods).

**Fig. 3:**
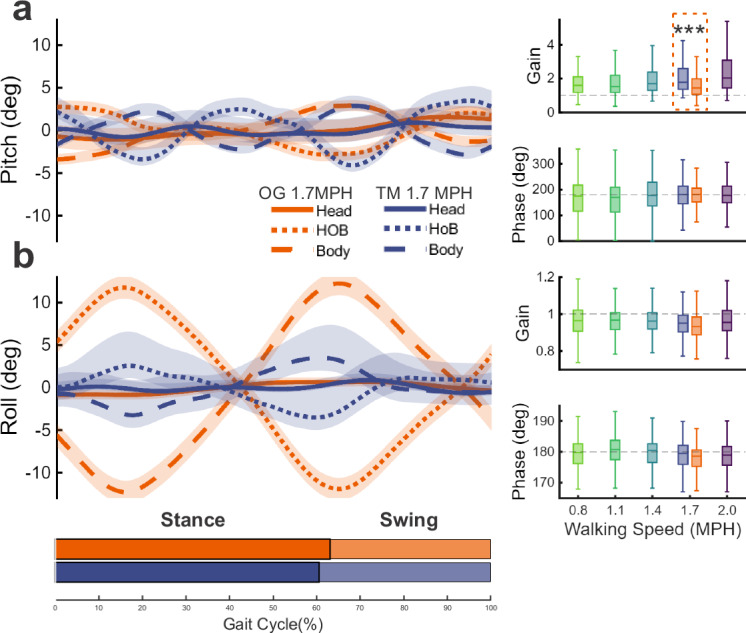
Head-on-body movements vary across axes and walking conditions. Cycle-averaged motion in pitch (**a**) and roll (**b**) axes for head-in-space (solid line), body-in-space (dashed line), and head-on-body (dotted line) are illustrated during treadmill walking at 1.7 MPH (blue), and overground walking (orange) in representative Monkey J. Horizontal bars along the bottom indicate the stance and swing phase for each condition. Right panels show the average head-on-body gain (top) and phase (bottom) for each condition from the data of all monkeys. Dashed gray lines indicate perfect compensation, i.e., gain of 1 and phase of 180 degrees. Notably, overground walking has better head-on-body compensation (gain) in pitch. Results are presented as boxplots, which show the median (center line), interquartile range (box), and whiskers extending to the nonoutlier (1.5 IQR) maximum and minimum. All *p* values are reported in Supplementary Tables 7 and 8 (***p < 0.001).

We first analyzed compensation during treadmill walking. Figure 3 illustrates this comparison between the pitch (Fig. 3a) and roll (Fig. 3b) axes, which exhibited the least and most effective compensation, respectively. Pitch motion displayed partial compensation, as the phase of head-on-body versus body-in-space motions was close to 180 degrees (Fig. 3a, bottom right panel), but the gain of this compensation was actually greater than unity, indicating overcompensation (Fig. 3a, top right panel). Ineffective compensatory head-on-body motion was also observed in vertical movement (Figs. S7a and S8). This inadequate compensation may partly reflect biomechanical constraints: vertical translation is mechanically coupled to head pitch^9^, thereby limiting the degrees of freedom available for independent head control. In contrast, roll exhibited near-ideal compensation with gain near 1 and phase near 180 degrees in all conditions (Fig. 3b, right panels) despite larger body-in-space motion. Further, effective compensation occurred in the lateral, fore-aft, and yaw axes (Fig. S7b–d, Fig. S8, Tables S7 and S8). The overcompensation observed in the pitch and vertical axes across speeds is consistent with their greater head-in-space oscillations. However, these axes diverged in how compensation changed with speed: pitch gain worsened, while vertical compensation improved (see Table S7).

We then compared these findings to overground walking at matched speeds. Phase remained close to 180° across axes (Figs. 3 and S5, orange triangles in the right panels; Table S8), indicating that compensatory timing was preserved. However, pitch gain improved significantly during overground walking—approaching unity—indicating stronger, more complete compensation and aligning with the enhanced head-in-space stabilization observed under overground conditions (Fig. 3a, right top panel, *p* = 3.92E-6; Fig. S8; Table S7). Gain and phase data of individual animals for each axis are illustrated in Fig. S9, and accompanying statistics are presented in Table S9.

### Motor unit recordings reveal phasic and context-dependent neck muscle recruitment

**Fig. 4:**
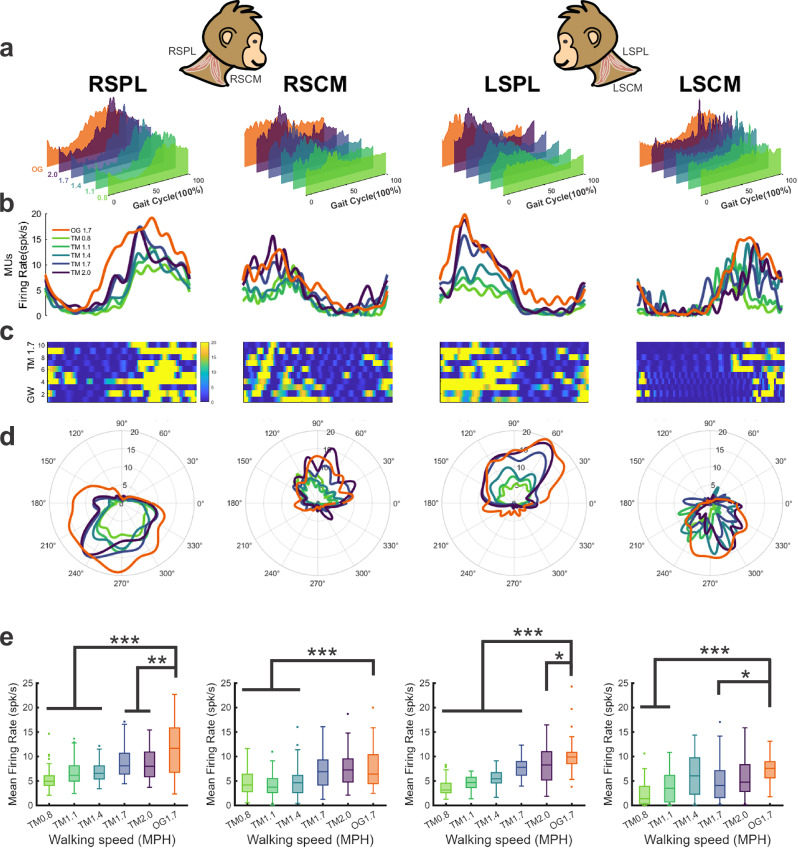
Neck muscle activity showed phase-dependent, reciprocal modulation during locomotion in all conditions. Average time course of multi-motor unit activity (**a**), average time course of single motor unit activity (**b**), and heatmaps of representative motor units across individual trials (**c**) demonstrate that each muscle showed modulation opposite to its contralateral pair (i.e., RSPL vs. LSPL) and its ipsilateral antagonist (i.e., RSPL vs. RSCM). Muscle activation showed a substantial increase with higher speed, while the activation phase in the same muscle is consistent across speeds (polar plots, **d**). **e** Mean firing rates of motor unit responses in all conditions for the SPL and SCM muscles over the stride cycle during locomotion across conditions. Notably, neck muscle responses were stronger during overground walking compared to treadmill walking at the matched speed, except for RSCM. Results are presented as boxplots, which show the median (center line), interquartile range (box), whiskers extending to the nonoutlier (1.5 IQR) maximum and minimum, and outliers (RSPL: *N* = 321; RSCM *N* = 281; LSPL *N* = 280; LSCM: *N* = 170). All *p* values are reported in Supplementary Table 10 (***p < 0.001, **p < 0.01, *p < 0.05).

Taken together, the above results emphasize that head stabilization performance is greater for overground than speed-matched treadmill walking. However, they do not provide direct insight into the motor control strategy that provides greater stabilization in the former case. To understand how this occurs, we next directly investigated motor unit activity using fine-wire EMG. Motor unit activity was recorded bilaterally from the SPL and SCM muscles during treadmill and overground locomotion using fine-wire electrodes, with the single motor unit activity sorted for analysis (Fig. 4). SPL and SCM are two key muscles that contribute to stabilizing the head and are accessible for recording^45,46^. These data were then normalized to the gait cycle as above, displaying stable activation phase-locked to the gait cycle both on average (Fig. 4a, b) and in individual strides (Fig. 4c). During treadmill walking, the average motor unit activity of all four muscles increased when speed was higher (Table S10). Furthermore, the response phase of each muscle remained constant across speeds and exhibited reciprocity with its contralateral pair and ipsilateral antagonist (Fig. 4d). For example, we found that the right SPL was activated from late stance to toe-on, while the right SCM was activated during early stance. Notably, this pattern of reciprocal phasic burst activity occurred between both bilateral muscle pairs and remained consistent across all speeds (see Fig. 4d).

We next compared the activation of these same muscles during overground walking with treadmill walking. Interestingly, motor unit responses during overground walking exhibited significantly higher activity compared to speed-matched (i.e., 1.7 MPH, blue trace) treadmill walking (Fig. 4e, *p* = 0.008 & 3.3E-4 for right & left SPL, and *p* = 0.03 for left SCM, Table S10). Muscle activity during overground walking even exceeded that observed during the highest treadmill walking speed (2.0 MPH, purple trace) in SPL muscles (Fig. 4e*p* = 0.004, 0.039, respectively; Table S10). However, we found that initiation of activity in the overground walking context typically preceded that observed during treadmill walking, most notably for the SPL muscles (Fig. 4d, orange traces versus other colors). This observation prompted us to consider whether these differences corresponded to a straightforward increase in activity or to an altered relationship between the neck muscles.

### Muscle activation structure is conserved across treadmill speeds but reorganized during overground walking

Head movement is controlled by the coordinated activity of all the muscles in the neck. Moreover, the temporal patterns of activation of these muscle groups over time represent a motor control strategy for executing the desired action. Specifically, we define a motor control strategy as the combination of 1) relative contributions of muscle activations to repeated groups of coordinated muscles, and 2) the recruitment sequences of these coordinated activity patterns over time. Flexible control of muscle activity involves scaling the same fundamental strategy to match the force required under different contexts. This scaling preserves the relative coordination structure, with activation magnitudes changing while the latent geometry remains similar. This geometric approach to defining a motor control strategy thus reduces to whether the paths in the muscle activation space have the same shape. A consistent activation structure (shape) across contexts, with potentially different scales, would suggest a conserved stabilization motor strategy, whereas the emergence of novel activation geometries across contexts would indicate the brain flexibly recruits different control strategies as a function of context.

Prior work has demonstrated that dimensionality reduction techniques can uncover temporal patterns of muscle coactivation during behaviors such as locomotion (factor analysis^21–23^; non-negative matrix factorization, NNMF^47–49^) and wrist or arm movements (principal component analysis, PCA^37,39,50^). Here, our goal was to compare coordination patterns across conditions. Thus, given that PCA’s signed loadings succinctly capture both synergistic and reciprocal relationships, we employed PCA to investigate the motor control strategy for head stabilization across conditions. Based on the individual motor unit recordings (Fig. 4), we hypothesized that different walking speeds would elicit the same control strategy. In PCA space, this hypothesis predicts that the muscle population exhibits trajectories with a similar geometry throughout the gait cycle, i.e., the path is continuously scaled in size with walking speed, with little distortion to shape. Such consistent activation geometry would suggest a conserved stabilization strategy, while novel geometries across contexts would indicate flexible recruitment of distinct strategies. However, we did not expect overground walking to share this strategy due to the observed changes in head stabilization and differences in mechanical forces and sensory inputs in self-driven locomotion.

To test our hypothesis, we first applied PCA within each condition to focus on the within-context activation structure. We observed qualitatively similar temporal patterns of recruitment in the muscle population across conditions, as illustrated by the cyclical shape of the gait-averaged PC activity in the space spanned by the top two PCs of each condition, which was expected given that the behavior is cyclic (Fig. S10a). We also found consistent relationships between muscle contributions to PC1 in each condition- specifically, the product of PC1 weights between every antagonistic muscle pair (e.g., right SPL vs right SCM or right SPL vs left SPL) is always negative (Fig. S10b). These represent that opposing muscle groups need to act in unison. Quantitatively, we computed path similarity scores (the best correlation under rigid rotation and translation; see Methods) across these within-condition PCA projections and found that this value remained above 0.79 across all treadmill speeds, with a consistent, gradual decrease between more different speeds (Fig. S10c, blue trace). Although overground walking showed relatively high path similarity (e.g., 0.81 compared to the matched treadmill speed), it did not follow the treadmill trend, in which similarity decreases monotonically with increasing speed differences. This observation indicates that its activation pattern is not simply a globally scaled variant.

**Fig. 5:**
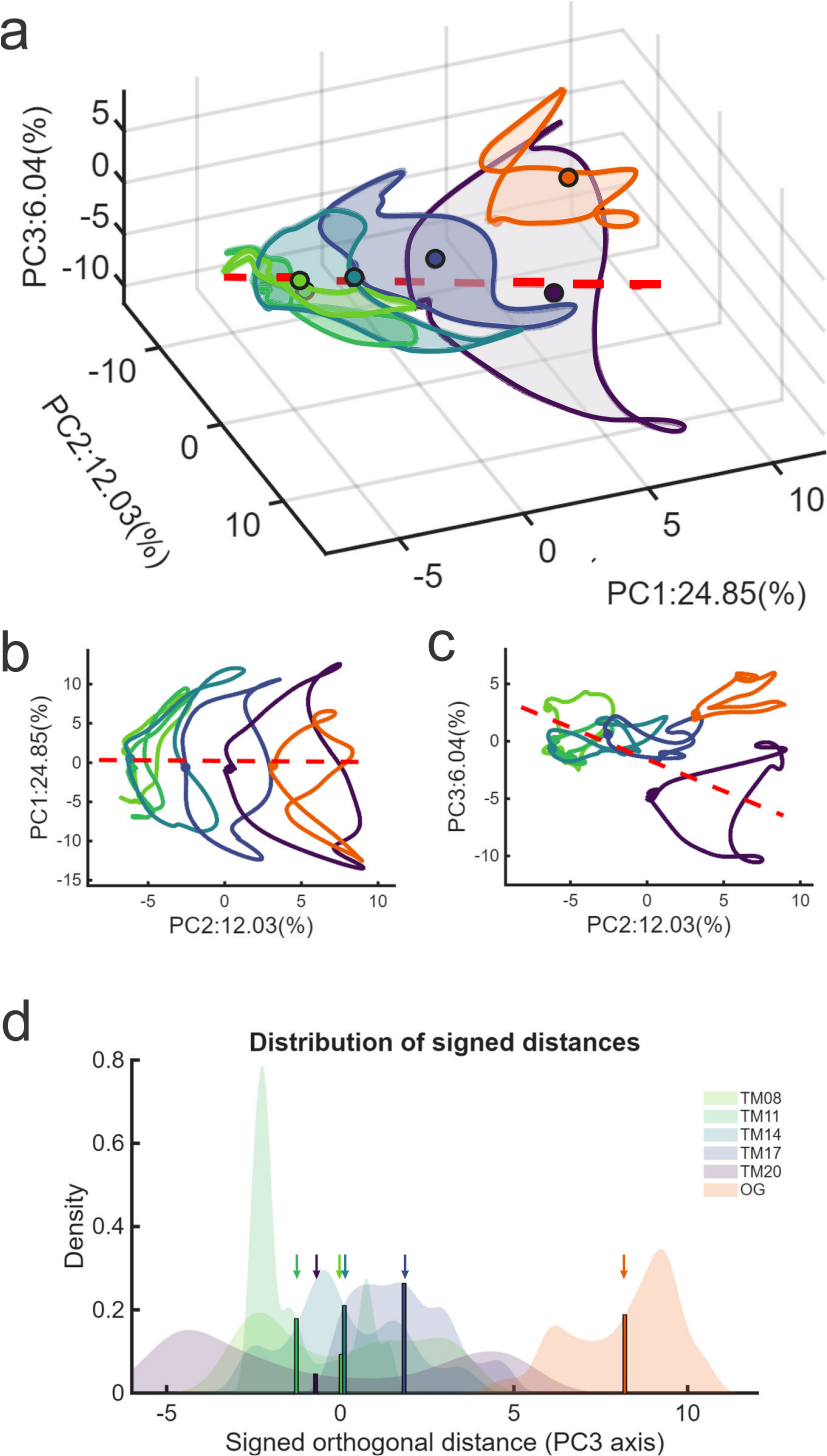
Population structure of neck muscle responses during walking. In the common PCA space, neck muscles exhibit speed-dependent translations and larger differences between ground and treadmill walking (**a**). Treadmill trajectories show smooth, speed-dependent translations along PC2 (**b**), whereas overground walking yields a distinct population geometry, separated along PC3 (**c**). Dots mark the trajectory center for each condition (**a**) or start of gait cycle (**b**, **c**); the black arrow is the linear fit through treadmill centers and points toward higher speeds. **d** Signed orthogonal distance distributions. Vertical lines mark the means of all conditions. Distances are residuals to the reference line projected onto PC3.

To further explore whether overground walking exhibited a different control strategy from treadmill walking, we computed a shared latent space by performing joint PCA across all conditions to better capture variance across contexts. Within this shared space, we computed a speed vector aligned with the axis of best fit across treadmill speeds in the top three principal components (Fig. 5a, see Methods). The muscle population trajectories across contexts further emphasize that treadmill walking at different speeds had scaled trajectories in the PC1 and PC2 space (Fig. 5b) while overground walking did not occupy the same space as the treadmill at the same speed (Fig. 5c). PC3 revealed an additional dimension of deviation for overground walking: rather than falling along the continuum of treadmill trajectories, the overground trajectory was displaced from the speed vector (Fig. 5c). We quantified this deviation by calculating signed orthogonal distances for each condition (Fig. 5d; see Methods), which confirmed that overground walking was distant from all treadmill speeds.

To verify our analyses were robust across dimensionality reduction methods, we also applied NNMF, a technique widely used in motor control studies to extract muscle synergies^47–49^, to the same neck-muscle EMG dataset. The resulting low-dimensional structures exhibited geometries similar to those obtained with PCA and reproduced the same key results: overground walking elicited muscle activation patterns that were notably displaced relative to treadmill walking. Specifically, the spatial patterns of Synergies 1, 2, and 4 closely resembled those derived from PCA, and the NNMF trajectory translated systematically with treadmill speed, indicating strong methodological consistency (Fig. S11a). The signed orthogonal distance analysis further confirmed an overground displacement relative to the treadmill conditions (Fig. S11b).

Taken together, our results indicate a systematic, context-dependent modulation of motor control strategies during locomotion. Specifically, a more consistent population-level muscle activation structure with smooth modulation enables head stabilization in response to changes in speed during treadmill walking. Overground walking, however, instead displayed a more distinct muscle activation structure, suggesting an altered coordination pattern of neck muscle activation. Given the improved stabilization of head motion in overground walking, the muscle population activity during overground walking may represent a more effective motor control strategy.

### Autonomic arousal enhances neck muscle recruitment without altering the head stabilization strategy

Thus far, we have observed that increasing muscle activation with speed during treadmill walking did not alter the underlying motor control strategy. However, changing the external behavioral context (overground versus treadmill walking) resulted in differences in both overall muscle activity and the specific activation structure at the population level. As previous work has established that internal state can modulate muscle activation (e.g., in lower limbs^51,52^), we next investigated how overall muscle activation and motor control strategies are affected by perturbing internal state using a social arousal paradigm (see “Autonomic arousal paradigm”, Methods). Notably, previous work in our group found that greater autonomic arousal significantly enhances the neck muscle responses to passively applied head motion^34^, suggesting that heightened autonomic arousal would enhance neck muscle activation and thus head stabilization during locomotion. However, based on the consistency in treadmill walking despite changes in muscle activation, we hypothesized that this internal change would not greatly impact the underlying control strategy, as the mechanical and sensory aspects of the context remained the same.

**Fig. 6:**
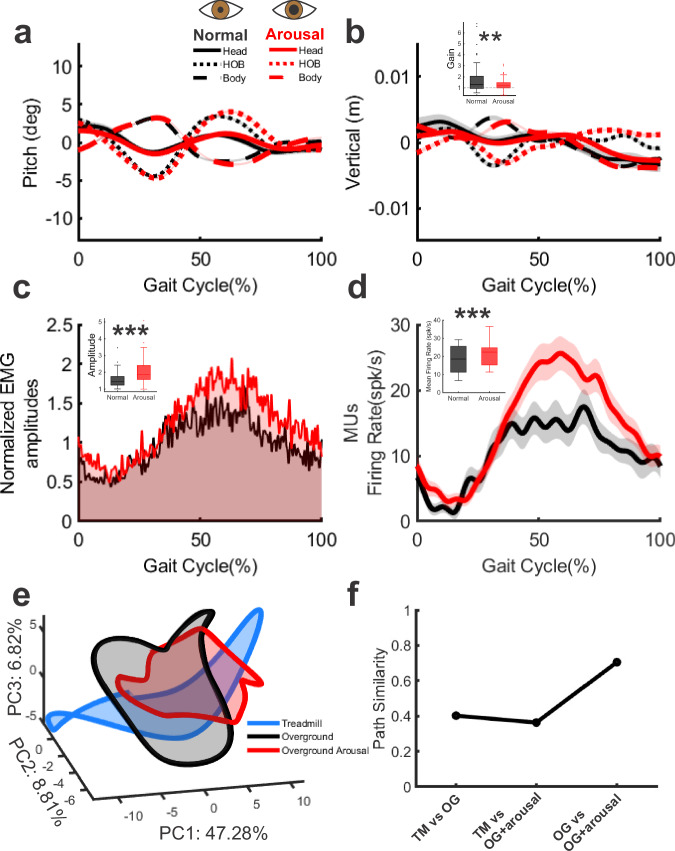
Autonomic arousal enhances neck motor unit responses with a mild change in activation pattern during overground walking. **a**, **b** Comparisons of averaged head-in-space (solid), body-in-space (dashed), and head-on-body (dotted) pitch and vertical movements across the gait cycle under normal (black) and arousal (red) conditions in representative Monkey B. Autonomic arousal generated improved compensatory head-on-body motion (inset bar plot; gain of vertical compensatory head-on-body motion; *N* = 140). RSPL muscle activity (**c**) and motor unit responses (**d**) during the gait cycle show enhanced peak activity under arousal than normal overground walking. Inset bar plots compare amplitude (*N* = 164) and firing rate (*N* = 74), respectively. **e** Muscle population activity during treadmill (blue), overground (black), and arousal overground (red) walking at 1.7 MPH is projected into a common PCA space (top 3 PCs). **f** Path similarity analysis in the top two PCs shows that locomotor propulsion mode (treadmill vs. overground) leads to larger differences in activation structure than arousal. Results are presented as boxplots, which show the median (center line), interquartile range (box), and whiskers extending to the nonoutlier (1.5 IQR) maximum, minimum, and outliers. All *p* values are reported in Supplementary Table 11, **p < 0.01, ***p < 0.001 (TM vs OG: 0.402; TM vs OG + arousal: 0.363; OG vs OG + arousal: 0.705).

Arousal was quantified by measuring the change in the subject monkey’s pupil size. We began by confirming that our paradigm resulted in pupil dilation compared with the control condition (*p* < 0.001). We next examined changes in head and body motion as well as in the associated neck muscle responses in the heightened arousal versus control conditions. Our analysis of head-in-space, body-in-space, and head-on-body movement is shown in Fig. 6a, b for pitch rotation and vertical translation and Fig. S12 for all other directions. Consistent with our hypothesis, we found that the head was more stable relative to space in the increased arousal conditions compared to the control condition (Fig. 6b; vertical amplitude *p* = 0.048; total variation *p* = 0.004; Fig. S12d; yaw: amplitude *p* < 0.001 and total variation *p* = 0.001, Table S11). Additionally, monkeys generated improved compensatory head-on-body motion during heightened arousal (Fig. 6b; vertical gain: *p* = 0.007; Fig. S12d: yaw gain: *p* = 0.029). Consistent with this latter finding, we likewise found that neck muscle activity was significantly enhanced during increased autonomic arousal (Fig. 6c, *p* < 0.001), while the timing of activity relative to the gait cycle (i.e., response phase) was unchanged (*p* > 0.05). Comparable results were obtained from our quantification of neck motor unit responses (Fig. 6d, *p* < 0.001).

To further understand how the control strategy for head stabilization is achieved across different contexts, we next asked whether neck muscle population activity displays a markedly altered geometry during locomotion in the increased arousal state. We performed joint PCA-based analysis of muscle activity, as in Fig. 5a, on the three speed-matched conditions: TM1.7, overground walking, and overground walking with heightened arousal. Figure 6e compares the structure of neck muscle population activity, with each condition projected into the space formed by the top three shared principal components (PC1-3). Overall, we found that the geometry largely remained intact during overground walking for both states of arousal (Fig. 6e, compare black with red; Fig. 6f, overground vs overground + arousal: 0.705). In contrast, the geometry during treadmill walking differed more (Fig. 6e, blue), showing substantially lower path similarity to the heightened as well as standard arousal overground walking conditions (Fig. 6f, path similarity: treadmill vs overground: 0.402; treadmill vs overground + arousal: 0.363).

Taken together, these results suggest the increase in motor unit activity observed during autonomic arousal does not substantially alter the underlying strategy used during overground walking, in contrast to the larger differences observed relative to treadmill walking.

## Discussion

A central question in motor control is whether the brain relies on a fixed coordination strategy, recruiting muscles in the same sequence and proportions across conditions, or whether it flexibly reorganizes motor output to meet changing behavioral demands. This study directly addressed that question by examining how the brain stabilizes the head, a fundamental behavior supporting gaze and balance, across distinct locomotor contexts. We found that, while head stabilization was achieved in both the treadmill and overground walking, it was more effective during overground locomotion at matched speeds. Quantification of head and body motion revealed improved stabilization measures and enhanced compensatory head-on-body movement across multiple axes during overground walking, particularly pitch, suggesting the engagement of a more refined motor strategy. To probe the control strategies underlying these kinematic differences, we analyzed neck muscle activity at both single-muscle and population levels. Although recruitment remained phase-locked across contexts, the population-level activation structure diverged: treadmill walking was governed by shared coordination patterns that scaled smoothly with speed, whereas overground locomotion evoked stronger muscle activity and a coordination strategy that diverged markedly, becoming displaced within the latent space. Notably, heightened arousal further amplified muscle recruitment while producing only modest alterations in the underlying activation geometry. Together, these findings directly demonstrate that motor coordination for head stabilization is not fixed but flexibly reconfigured by the brain according to behavioral context.

### Overground locomotion recruits altered sensorimotor strategies for head stabilization

Our results show that head stabilization relative to space is greater during overground than speed-matched treadmill walking in primates. Previous quadrupedal studies have focused on treadmill locomotion (e.g., nonhuman prmates^7,9^; horses^18^) and reported improved head stabilization with increased speed^9,18^, consistent with our results. The enhanced head stabilization we observed during overground walking raised key questions about whether the brain uses a common motor strategy for head stabilization across contexts or flexibly reconfigures coordination to meet situational demands. Although the overground track was wider than the treadmill belt, animals consistently walked near the center of both surfaces, making it unlikely that this difference meaningfully affected step width or stabilization. Our neck muscle recordings revealed robust, phase-locked activation across conditions. For instance, SPL activity peaked during ipsilateral hindlimb swing, consistent with prior findings in horse^17^, whereas SCM activity peaked during ipsilateral hindlimb stance, resulting in a consistent overall pattern in which contralateral SPL-SCM pairs were coactivated. All neck muscle activity scaled with treadmill speed, but timing and coordination remained consistent, paralleling stable limb activation patterns reported in primates^53–55^. Notably, unlike limb muscles—which show greater activation on treadmills^56–58^ in humans—neck muscles were more strongly recruited during overground walking (Fig. 4e). Enhanced recruitment likely supports the consistent or even improved head stabilization observed at higher speeds and under overground conditions (Fig. 2). Importantly, as further discussed below, we identified that muscle population activity varied systematically across contextual changes—including speed, propulsion mode, and autonomic arousal to different degrees—indicating that motor control engages a flexible strategy to maintain head stabilization.

### Context reorganizes the latent structure of muscle population activity during locomotion

To determine whether motor coordination strategies differ across contexts, we then analyzed temporal patterns of muscle population activity using PCA. While PCA is often used for dimensionality reduction, it also provides a powerful framework for reorienting high-dimensional data to reveal the primary modes of coordination amongst data features—here taken as muscle activations over time. We extracted trajectories taken through PCA space to determine the structure of muscle recruitment dynamics, following work on the geometry of neural and muscle population activity^35–39,50,59,60^. Prior studies seeking to understand multi-muscle coordination have often applied NNMF^47–49^, which is mathematically well-suited because EMG amplitudes and motor unit firing rates are inherently non-negative. Here, our goal was to capture and compare coordination patterns across conditions, for which PCA’s signed loadings provide a compact description of both synergistic and antagonistic relationships. Consistent with this distinction, because NNMF is applied to non-mean-centered data, we introduced an additional “offset-like” synergy that captured baseline or amplitude shifts across conditions (Synergy 4, Fig S11a), thereby enhancing the separability between conditions. This difference arises from preprocessing assumptions underlying each approach (PCA’s mean-centering versus NNMF’s non-centered, nonnegative decomposition). Importantly, when we applied NNMF to the same dataset, the resulting synergy activations closely paralleled the PCA-derived patterns, confirming the robustness of the PCA-based findings.

Crucially, work applying dimensionality reduction to behavioral or muscle activity data has broadly been limited to comparisons along individual dimensions^19,22–25,27,28,61^ (i.e., PC1 in context 1 vs PC1 in context 2), neglecting potential interactions among components. Developing approaches have sought to quantify multidimensional relationships in latent space^45,46,57^, including during both human^62^ and animal^63^ locomotion. We advance this work by systematically quantifying how locomotor behavior across a broader range of contextual changes reshapes the geometry of neck muscle population activity.

Our analysis revealed that muscle activity trajectories exhibited a clear rotational structure in the first two principal components, reflecting the rhythmic cadence of locomotion, while subsequent dimensions captured non-rotational dynamics likely associated with nonlinear processes such as movement initiation, termination, postural stabilization, and feedback control^36^. During treadmill walking, this population geometry scaled smoothly with speed: the size of the rotational trajectory increased, while its shape remained similar (Fig. 5b). This suggests that, where possible, the brain flexibly adapts its control strategy to varying task demands (e.g., increasing velocity) by modulating the amplitude of recruitment rather than altering coordination. These findings are consistent with prior reports showing velocity-dependent scaling of low-dimensional trajectories at the neural^50^, muscular^22^, and kinematic^62^ levels, as well as translational shifts in latent space observed in muscle and neural populations^37,50^. We thus conclude that, when task demands are comparable, the brain preserves a more similar control strategy and accommodates changes by scaling activation rather than engaging in a full reorganization of coordination.

Importantly, however, this scaling was context-dependent and did not extend uniformly across conditions. In contrast to treadmill walking, overground locomotion exhibited a more substantially restructured organization of muscle population activity, suggesting engagement of a more effective motor control strategy. While previous work has shown that population trajectory structure can shift during complex movements at both neural^64,65^ and muscular^23^ levels, our findings provide the first direct evidence that even unconscious behaviors like head stabilization during locomotion are governed by flexible strategies that depend crucially on conditions. Specifically, we observed more sustained peaks in motor unit firing (Fig. 4) and a latent geometry that was separable from all treadmill speeds (Fig. 5a–c). These differences likely reflect both biomechanical factors as well as the enriched sensory inflow available during overground locomotion, which preserves the normal coupling between motor efference and multisensory feedback (optic flow, proprioception, vestibular cues) as the animal moves through three-dimensional space^66^. We speculate that such multimodal integration supports a more efficient stabilization strategy by better damping locomotor-induced forces that would otherwise disrupt head position. Consistent with this, we observed lower head movement variation during overground walking, even compared with the slowest treadmill speeds (Fig. 2).

These findings demonstrate the principle of context-conditioned variability, i.e., that the same muscle activation can yield different movement outcomes as conditions change^67,68^. In our study, increased muscle recruitment in two contexts (overground walking and higher treadmill speeds) elicited divergent motor control strategies, which were accompanied by different stabilization performance, illustrating the need for adjustments in coordination strategy when context shifts to achieve the same behavioral goal. More broadly, the context-dependent reorganization of control strategies we uncover may help explain clinical phenomena such as why patients with peripheral vestibular sensory loss often exhibit impaired postural but preserved movement control^14^.

### Autonomic arousal amplifies muscle recruitment without altering core motor strategy

It is well established that motor control can be influenced by internal brain states such as arousal^34,51,52^. Our findings expand on this work by demonstrating that autonomic arousal during locomotion leads to increased neck muscle recruitment and reduced head movement in space, demonstrating the role of the autonomic nervous system in enhancing the output of descending pathways at the motor unit level. Notably, while overall activation increased, the structure of population-level muscle activity remained largely unchanged: the geometry of latent muscle activity during high-arousal overground walking more closely resembled that of the non-aroused overground condition despite increased recruitment. This indicates that differences in control strategy between treadmill and overground walking do not arise merely from changes in the state of arousal or overall muscle activity level or overall muscle activity level. In summary, autonomic arousal did not produce a substantial shift in motor control strategy during overground walking; instead, mechanical demands and the available motor efference and sensory information exerted the predominant influence. Enhanced activity during heightened autonomic arousal likely improves stabilization in challenging or unpredictable situations that require precise balance and coordination by supporting head control and facilitating the reliable integration of visual and vestibular cues.

### Context-specific motor strategies for head stabilization: principles for motor control

A central question in motor control research is whether the brain relies on a universal control strategy, recruiting the same muscles in the same sequence at proportional levels to drive a given behavior, or instead flexibly adapts to the demands of each context. Head stabilization during locomotion is critical for sensorimotor function, ensuring a stable platform for visual and vestibular processing that supports gaze control, spatial orientation, and interaction with the environment. Here, we provide direct evidence that the brain reconfigures muscle coordination strategies to maintain head stabilization across distinct locomotor contexts. By recording activity across multiple neck muscles, we show that the nervous system dynamically coordinates muscle recruitment to maintain postural control under varying task demands. During treadmill walking, we observed increased muscle recruitment and smooth scaling of latent motor patterns with speed, consistent with a stable control strategy that adjusts to biomechanical demands. In contrast, overground walking elicited markedly different activation patterns and enhanced head stabilization, suggesting the recruitment of a potentially more effective strategy. To our knowledge, this is the first direct demonstration of flexible, context-specific neck muscle coordination supporting head stabilization. These insights highlight how different locomotor contexts can substantially modify motor control strategies through distinct mechanisms. We suggest future locomotor studies in humans, which have primarily focused on torso and lower limbs^69,70^, expand to more thoroughly consider head and upper-body control. Finally, our results parallel principles used in robotics, where complex systems are simplified into context-specific control regimes^71–73^. Similarly, rather than employing a single motor control strategy to accommodate all circumstances, the nervous system selects among low-dimensional, context-dependent strategies and smoothly adapts them to specific task demands.

## Limitations and future directions

This study provides new insight into how the primate nervous system flexibly coordinates neck muscle activity to stabilize the head across diverse locomotor contexts. However, several limitations warrant consideration. Our subject cohort comprised three rhesus macaques for kinematic analyses and two for bilateral neck EMG recordings, reflecting the ethical and practical constraints of non-human primate research. While this constrained our statistical power, robust within-animal effects and consistent trends across subjects provided strong internal validation. Further, to minimize potential confounds in acceleration-related measures, initiation and termination steps during overground walking were excluded. Extending recordings to longer tracks or more naturalistic environments could reveal how coordination adapts across continuous self-paced locomotion. Moreover, while the present results reveal distinct contributions of mechanical and internal-state influences on coordination, future studies leveraging self-driven or variable-resistance treadmills alongside controlled optic-flow perturbations could systematically dissociate the effects of control mode, mechanical load, and sensory feedback. Together, these directions would build on the present findings, which establish a foundation for understanding flexible motor coordination in naturalistic settings and illustrate the remarkable capacity of the motor system to adapt its control strategies across behavioral and sensory contexts.

## Methods

Three monkeys (Macaca mulatta, one female and two males) were used in this study. All experimental procedures were approved by the Johns Hopkins University Animal Care and Use Committee, which is accredited by the Association for the Assessment and in compliance with the guidelines of the United States National Institutes of Health (PR22M342). We have complied with all relevant ethical regulations for animal use. As we have described previously^74^, each animal was anesthetized and equipped with a titanium post fastened to the skull using titanium screws and dental acrylic to allow immobilization of the head and securing recording hardware. All animals recovered for at least 2 weeks before any experiments began.

### Data collection and analysis

#### Kinematics

All monkeys were trained for treadmill and overground walking quadrupedally (Fig. 1). Each animal was equipped with a collar that could be fixed to a custom primate chair suspended beneath a linear track, maintaining the animal on the walkway or treadmill while allowing relatively free behavior as well as video recording of their movements. The animals’ gaze, head, and body were otherwise unconstrained. All experiments were performed in the same room and with the same arrangement of equipment to ensure consistent visual and spatial context across sessions. During treadmill walking, animals walked on a motorized treadmill with a 16-inch-wide belt positioned on top of the overground walkway. Both surfaces were sufficiently wide to avoid constraining stride width and consisted of a thin rubber layer over a flat metal base, providing comparable stiffness and compliance. All monkeys were trained to walk at a range of different speeds (speed: 0.8, 1.1, 1.4, 1.7, and 2.0 MPH). One treadmill walking session consisted of 15–20 s of continuous walking at each speed. At least 10 successive gait cycles were extracted from each speed during a session. For overground walking, monkeys were trained to walk on a track (walking surface: 140 by 20 inches) at an average speed of 1.7 MPH. The speed of overground walking was calculated as the average length of the walking surface divided by the walking duration. The ends of the track were each mounted on a pivot that, when released, allowed the experimenter to rotate the monkey by 180° so that they could perform repeated traversals of the track. The monkey performed at least five round-trip sessions along the track during each session, which were pooled across sessions to compute kinematics and motor unit measures. To minimize the influence of transient fore-aft accelerations associated with gait initiation and termination, the first and final steps of each overground walking trial were excluded from analysis. We collected at least 100 strides from each monkey for each treadmill speed and for overground walking. Each animal was trained for a minimum of 2 months before any locomotor data were collected. Animals received preferred food items (dried fruit, nuts) or juice as positive reinforcement between walking bouts to encourage consistent locomotion at the target speed (1.7 MPH).

Four high-speed video cameras (Blackfly S BFS-U3-13Y3M, Teledyne FLIR) filmed from the sides (sagittal plane) to record the limb movement (100 Hz) (Fig. 1a). To ensure synchronization, frame capture was triggered by a hardware pulse, which was also recorded (OmniPlex, Plexon) for later alignment with analog data. DeepLabCut, an open-source deep learning software package enabling markerless pose estimation, was then used to extract the animals’ 3D posture (x, y, z coordinates) for gait analysis^75^. The 3D positions of the bilateral shoulder, elbow, wrist, metacarpophalangeal, hip, knee, ankle, and metatarsophalangeal joints were extracted. These data were used to calculate 3D joint angles and divide gait cycles. One gait cycle was defined as the time interval between two successive paw contacts of the right hindlimb, while the onset of the swing phase was set at the start of the forward movement of the right hindlimb^76,77^.

Two additional cameras synchronized to those described above were mounted on the chair to separately track the head and body positions. This was achieved using an open-source, marker-based tracking system^78^ in which visual targets equipped with retroreflective markers were attached to the head implant and the thoracic spine of the animal. Both the head and body were treated as rigid bodies capable of rotation and translation in space. Head-on-body movements were then calculated by analyzing the relative positions between the head and body. Additionally, a head-mounted 6D inertial measurement unit (Model 634, TE Connectivity) recorded linear head acceleration and rotational velocity.

The space-fixed coordinate frame (x, y, z) served as the origin for all other coordinate frames, defining the x-y (horizontal plane), y-z (sagittal plane), and x-z (coronal plane) planes (Fig. 1a). The x-axis (lateral) pointed positively to the monkey’s left, the y-axis (fore-aft, FA) aligned with the forward walking direction, and the z-axis (vertical) was oriented positively downward. The retroreflective targets positioned on the head and spine defined the positions of the head and body in space.

The head-fixed coordinate frame (X_H_, Y_H_, Z_H_) was defined with X_H_ parallel to the internal-aural axis (pitch), Y_H_ parallel to the naso-occipital axis (roll), Z_H_ was normal to the X_H_-Y_H_ plane (yaw). The body-fixed coordinate frame (X_B_, Y_B_, Z_B_) was defined with X_B_ parallel to the transverse axis (pitch), Y_B_ parallel to the long axis of the trunk (roll), and Z_B_ normal to the X_B_-Y_B_ plane (yaw). Axes and positive rotations in all frames followed the right-hand rule.

Postural data obtained from markerless keypoint tracking were processed with MATLAB R2023a (MathWorks) and were smoothed by a 10 Hz low-pass filter and interpolated to a time base of 2000 points per gait cycle^77^. Average data were computed across all gait cycles within each condition for each animal. Toe-on and toe-off events were expressed as a percentage of gait cycle duration. To evaluate head-in-space stabilization, amplitude and total variation (*V*_Total_) were calculated. Amplitude was defined as the peak-to-trough difference, while total variation was computed as the sum of absolute differences between consecutive data points, expressed mathematically as:

$${{{{\rm{V}}}}}_{{{{\rm{Total}}}}}=\mathop{\sum }_{{{{\rm{i}}}}=1}^{{{{\rm{N}}}}-1}|{{{{\rm{X}}}}}_{{{{\rm{t}}}}+1}-{{{{\rm{X}}}}}_{{{{\rm{t}}}}}|$$

where *N* is the number of data points, ${X}_{t}$ is the measured value at each time point. To analyze the head-on-body coordination, we calculated the head-on-body gain and phase using the Hilbert transform. First, we derived the amplitude and phase of both the head-on-body signal and the body-in-space signal. The gain was then computed as the ratio of the head-on-body amplitude to the body-in-space amplitude. The phase was determined from the difference between the instantaneous head-on-body and the body-in-space phases.

#### Neck motor unit activity

Single- and multi-motor unit activity was recorded bilaterally using acutely-inserted, sterilized fine-wire electrodes in the SPL and SCM muscles in two monkeys (one female and one male). The electrodes consisted of a pair of stainless-steel wires (Stablohm 800 A; California Fine Wire) tightly wound together. The skin over the neck muscles was shaved and cleaned with isopropyl alcohol. Insertion was guided by ultrasound (SonoSite MicroMaxx, FUJIFILM Sonosite) under aseptic conditions. Single- and multi-motor unit data were recorded simultaneously with kinematic data collection. Muscle activity was amplified (×1000), bandpass filtered (30–10,000 Hz) (NeuroLog, Digitimer), and digitized at 40 kHz by a neural recording data acquisition system (OmniPlex, Plexon). Data were processed with MATLAB R2023a (MathWorks). Single motor unit activity was sorted from the EMG recording through a custom GUI. Raw EMG signals were full-wave rectified and bandpass filtered (100–10,000 Hz), then down-sampled to 1000 Hz. Following this, the data were smoothed by a low-pass filter (5 Hz) and linearly time-interpolated over a time base of 2000 points per gait cycle^77^. The EMG signals were normalized by the mean amplitude recorded at a treadmill speed of 1.7 MPH for each session and then averaged across sessions. Amplitude was calculated as the root mean squared value of the signal for each cycle.

### Principal components analysis

Following approaches used in studies of central motor control^35–40^, we applied principal components analysis (PCA) to datasets consisting of the normalized EMG. To calculate the subspace that explains response variance, EMG across all animals was combined into a single matrix. This matrix has dimensions of *T* × *n*, where n is the product of the number of gait cycles and muscles (50 gait cycles × 4 muscles = 200), and *T* equals 2000 time points (one gait cycle) multiplied by the number of conditions being analyzed (*T* = 2000 for a single condition and 2000 × [5 treadmill speeds + overground] = 12,000 for combined conditions). For the resulting *T* × *n* matrix, each column was first z-normalized. PCA was next applied to the normalized matrix via singular value decomposition, yielding principal component coefficients (PCs), component scores (reduced-dimensional version, X), and explained variances. The resulting matrix represented the projection of muscle activity onto the global principal components, which captured the dominant patterns of muscle activation in a compact, low-dimensional space. Path similarity measures were then computed to quantify the degree of trajectory shape change in the space, defined by the top 6 principal components, relative to a reference speed. Trajectories were mean-centered and aligned via rigid rotation using singular value decomposition to minimize Euclidean distance, and similarity was quantified as the coefficient of determination (*R*²) between the reference and rotated trajectories^50^.

To quantify differences in the trajectory positions within the shared PCA space while controlling for the effect of speed, we calculated distances between the points of each trajectory and a defined ‘speed vector’. Specifically, we first computed each trajectory’s center as the mean of its 3D points for each condition. To obtain an axis summarizing the progression from low to high treadmill speeds, we fit a line through the centers of the five treadmill speeds. For example, the matrix C was a 5 × 3 matrix, which included the centers of all five treadmill trajectories, *μ* = mean(C), and D = C-μ. This ‘speed vector’ *v* was the first right singular vector calculated by singular value decomposition of D, with its origin at μ and positive direction aligned to point from speed 0.8 toward speed 2.0. The arrow in Fig. 5a–c was drawn from μ along *v* with a length set by the span of the scalar projections D*v*. To quantify the deviations relative to this line, we projected each sample onto *v* and the residual onto a single orthogonal axis parallel to PC3. Specifically, we defined the positive unit vector:

$$q=({PC}3\,-\,({PC}3\cdot v)v)/({||PC}3\,-\,\left({PC}3\cdot v\right){v||})$$

with residual *r* = (X-μ)-[(X-μ)⋅*v*]*q*,and the signed orthogonal distance *d* = *r*⋅*q*. For each condition (2000 time points of PC123 trace), we calculated the kernel density estimate of *d*. This reports the distribution of condition-wise deviations in the plane orthogonal to the reference line, while fixing the sign according to the PC3 axis.

#### NNMF for EMG synergies

To confirm that our results were robust across dimensionality reduction methods, we further applied NNMF to our data for comparison. First, we constructed a non-negative EMG matrix (V) consisting of the normalized EMG (four muscles, 50 trials each) recorded under six locomotor conditions (TM0.8, TM1.1, TM1.4, TM1.7, TM2.0, OG1.7), consistent with our dataset for PCA. Data were normalized to the maximum within each gait cycle to yield *Vn*. We then obtained a rank-4 decomposition, *Vn* = W*H, where columns of W are muscle synergies and rows of H are their time-varying activation coefficients^8,47,79^. This approach and PCA share an assumption of fixed weights or loadings from the data onto the latent space. For visualization, synergies were smoothed within each condition using a short moving-median prefilter followed by a Savitzky–Golay filter (3rd order; window length 81), and the resulting coordinates were plotted as a 3D trajectory.

### Autonomic arousal paradigm

A social paradigm was used to investigate the impact of autonomic arousal on neck muscle modulation during locomotion. As a non-luminance mediated pupil size change is an established indicator of arousal^80–82^, we recorded pupil size to investigate the potential impact of increased arousal on head stabilization. The left eye was recorded using video-oculography (Firefly S, Teledyne FLIR) at 200 Hz as each monkey walked both with and without the presence of another monkey. Arousal levels were quantified based on changes in pupil size, measured during periods when the eye was relatively centered in the orbit (within 10° from the vertical and horizontal center), and normalized to each monkey’s baseline. In all cases, pupil size increased when the test monkey was in the presence of another monkey. Comparisons were then made between conditions for head motion, neck motor unit activity, and population dynamics.

### Statistics and reproducibility

#### LMEs modeling

We analyzed data from three monkeys, each contributing 100 strides per condition across six locomotor conditions (five treadmill speeds and overground), yielding 1800 strides in total. Biological replicates were individual monkeys; technical replicates were strides. All principal findings were observed in all three animals. To assess the effect of walking condition on kinematics while accounting for between-animal differences, we fit LME models with condition as a fixed effect (six levels, reference-coded to the 0.8 MPH treadmill condition) and a random intercept for subject^83^. Fixed-effect significance was evaluated from the fitted LME. Pairwise post hoc comparisons were performed with Holm–Bonferroni multiplicity correction.

$${{{\rm{Y}}}} \sim 1+{{{\rm{Condition}}}}+\left(1{{{\rm{|Subject}}}}\right)$$

where *Y* is a particular kinematic output measure (amplitude, total variation, head-on-body gain, or head-on-body phase). Categorical predictors were reference-coded with 0.8 MPH treadmill walking as the baseline level. Models were estimated by restricted maximum likelihood (REML) in MATLAB 2024a, with degrees of freedom estimated using the residual method. We report estimated marginal means (EMMs) with 95% confidence intervals (CIs) derived from the fixed-effects covariance for each condition. For all condition pairs, we enumerated contrasts to produce a comprehensive summary of estimates, standard errors (SEs), and CIs. For post hoc all-pairs comparisons, *p* values were adjusted using the Holm–Bonferroni procedure across the full set of pairwise tests. Statistical tests were two-sided with *p* = 0.05. Results are presented as EMM with 95% CI and (adjusted) *p* values for Supplementary Fig. 2, 5 and 8. In Figs. 2, 3 and Supplementary Figs. 1, 3, 4, 6, 7, and 9 results are presented as boxplots, which show the median (center line), interquartile range (IQR) (box), and whiskers extending to the nonoutlier maximum and minimum (1.5× IQR distance).

#### Additional statistical analysis

Distributional properties were assessed using the Lilliefors test, followed by either a *t* test or Wilcoxon rank-sum test as appropriate, to compare the mean overground speeds between three monkeys (*N* = 100 for each monkey), mean motor unit firing rates across contexts (RSPL: *N* = 321; RSCM *N* = 281; LSPL *N* = 280; LSCM: *N* = 170). For individual monkey’s statistics, data are presented in box plots containing median, interquartile range (IQR), and individual plots for outliers >1.5× IQR distance from the median. Within-subject linear regression analysis was performed to identify significant relationships between treadmill speed and the amplitude, total variation, gain, and phase of head-on-body movements (*N* = 100 for each monkey). Within-subject distributional properties were assessed using the Lilliefors test, followed by either a *t* test or Wilcoxon rank-sum test as appropriate, to compare the amplitude, total variation, and head-on-body movement (gain and phase) between treadmill and overground walking at matched speeds (*N* = 200 for each monkey). Statistical analyses were conducted using MATLAB R2024a (MathWorks), and a significance threshold of 0.05 was applied for all statistical tests. Raw data for all monkeys were provided in Supplementary Data 1.

### Reporting summary

Further information on research design is available in the Nature Portfolio Reporting Summary linked to this article.

## Supplementary information

Supplementary information

Description of Additional Supplementary Files

Supplementary Data 1

Reporting Summary

## Data Availability

All data supporting the findings of this study are available within the paper and its Supplementary Information. Additional raw data are available from the corresponding author upon reasonable request.

## Code availability

Custom MATLAB scripts used to generate the figures are available from the corresponding author upon reasonable request, no original code report.
