# Supplementary material for: Locomotion engages context-dependent motor strategies for head stabilization in primates
Source: Commun Biol. 2026 Jan 12;9:234. doi: 10.1038/s42003-026-09512-2 (PMC12901029; doi:10.1038/s42003-026-09512-2)
Supplement: Supplementary file 1 — Supplementary information [file 42003_2026_9512_MOESM1_ESM.pdf]

# Supplementary information

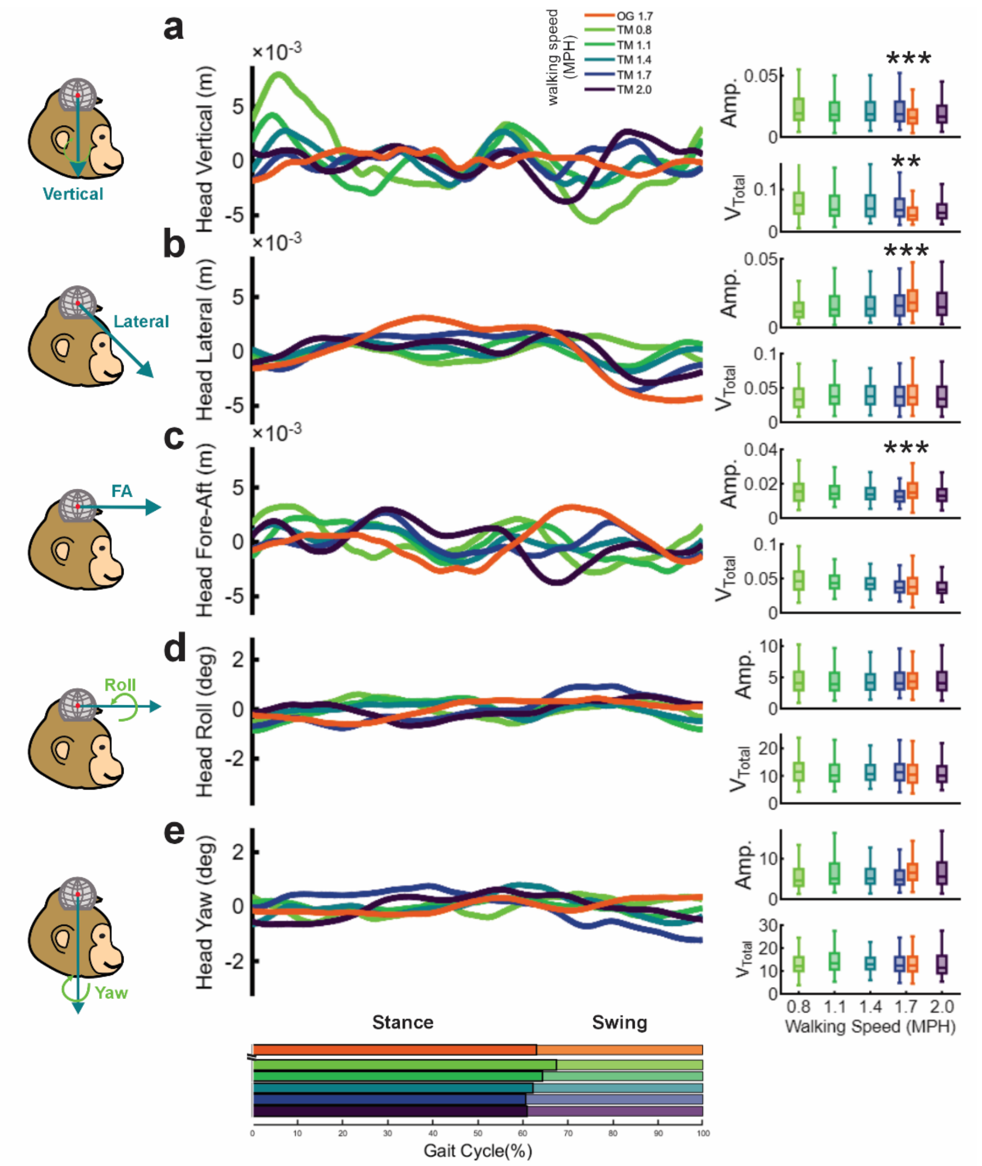


**Supplementary Figure 1:** Head-in-space rotational and translational positions across the gait cycle. Illustrations to the left indicate the axis being plotted. Time course of averaged head vertical (a), lateral (b), fore-aft (c), roll (d), and yaw (e) positions throughout the gait cycle in representative Monkey J, as in Figure 2b. Horizontal bars along the bottom indicate the stance and swing phase for each condition. The top and bottom panels to the right of each axis display Amp. and $V_{Total}$ for each condition from the data of all monkeys. Notably, roll and yaw position showed no significant differences across treadmill speeds, except that roll $V_{Total}$ TM 0.8 > TM 2.0 (p=0.0177). Fore-aft

position amplitude was lower at TM 1.7 than at TM 0.8 or TM 1.1 ($p=0.0146$, 0.0053, respectively), and $V_{Total}$ was lower at higher speeds except at adjacent speeds (all $p<0.042$). Lateral position was consistent except that TM 0.8 amplitude was below TM1.1, TM 1.7, and TM 2.0 ($p=0.0358$, 5.58E-4, 5.16E-6), and $V_{Total}$ was less than TM 1.1 and TM 1.4 ($p=5.54E-4$, 0.009). Results are presented as boxplots, which show the median (center line), interquartile range (box), and whiskers extending to the nonoutlier (1.5 IQR) maximum and minimum. Corresponding p values are reported in Supplementary Tables 1, 2.

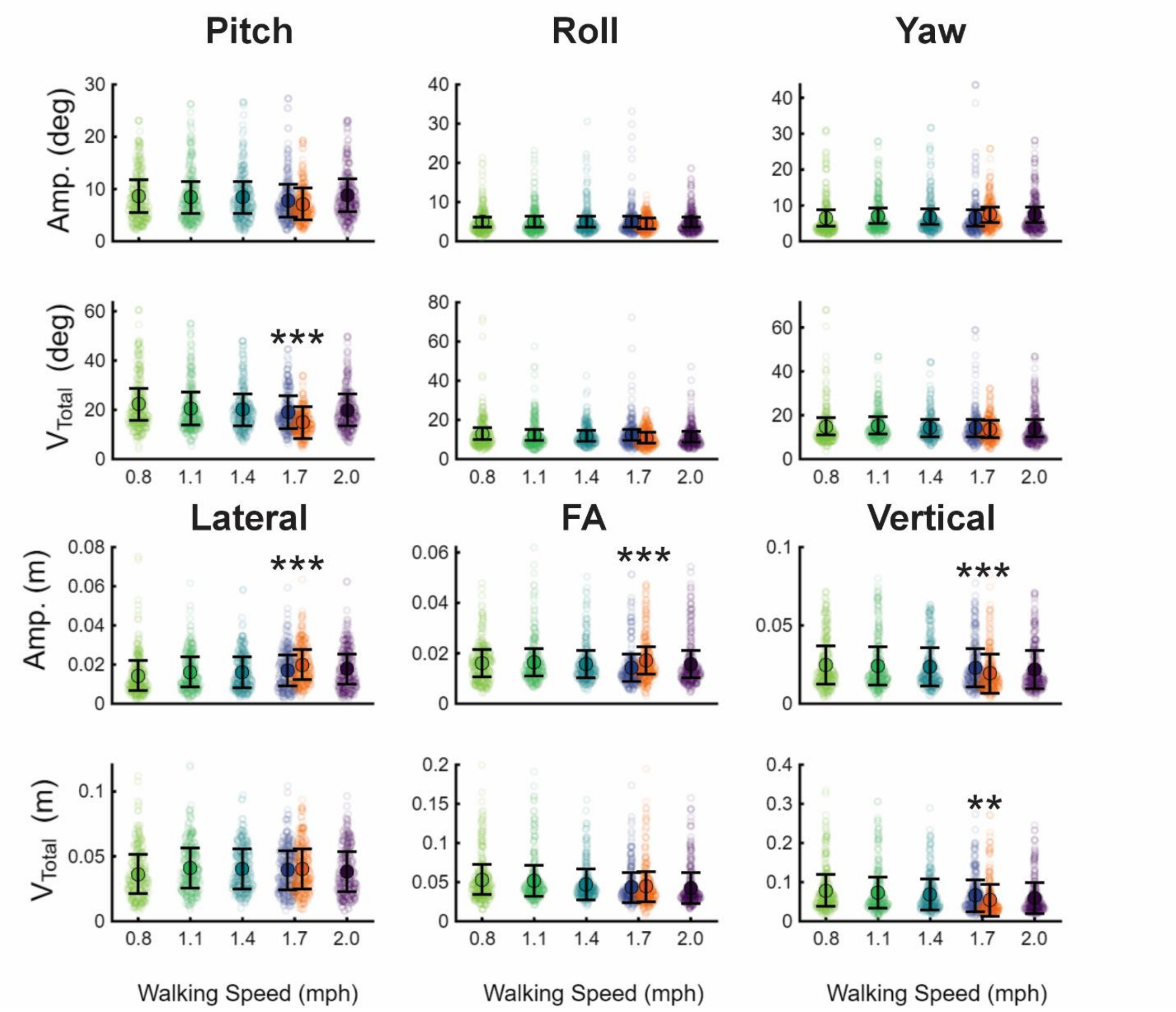


**Supplementary Figure 2:** Head-in-space rotational and translational position measures for all monkeys. For each axis, Amp. and $V_{Total}$ are shown as dot plots with LME-estimated means and 95% CIs. All corresponding p values are reported in Supplementary Tables 1, 2.

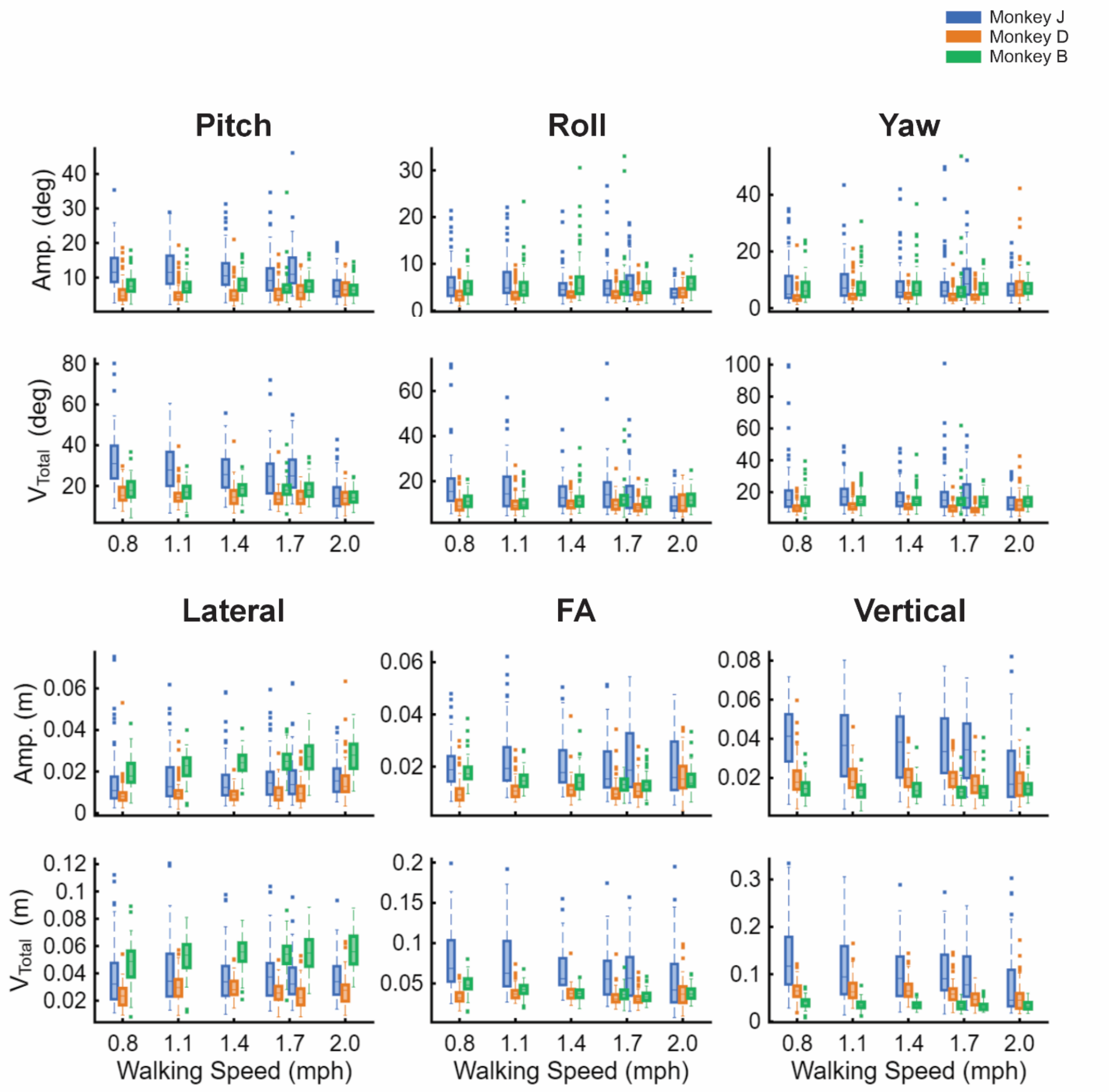


**Supplementary Figure 3:** Head-in-space rotational and translational position measures for each monkey. Amp. and $V_{Total}$ are shown separately for each animal in each axis (blue: Monkey J, orange: Monkey D; green: Monkey B). Monkeys exhibited similar trends across most axes. Linear regressions were applied to treadmill walking data when condition effects were significant. Results are presented as boxplots, which show the median (center line), interquartile range (box), and whiskers extending to the nonoutlier (1.5 IQR) maximum, minimum, and outliers. All corresponding r and p values are reported in Supplementary Table 3.

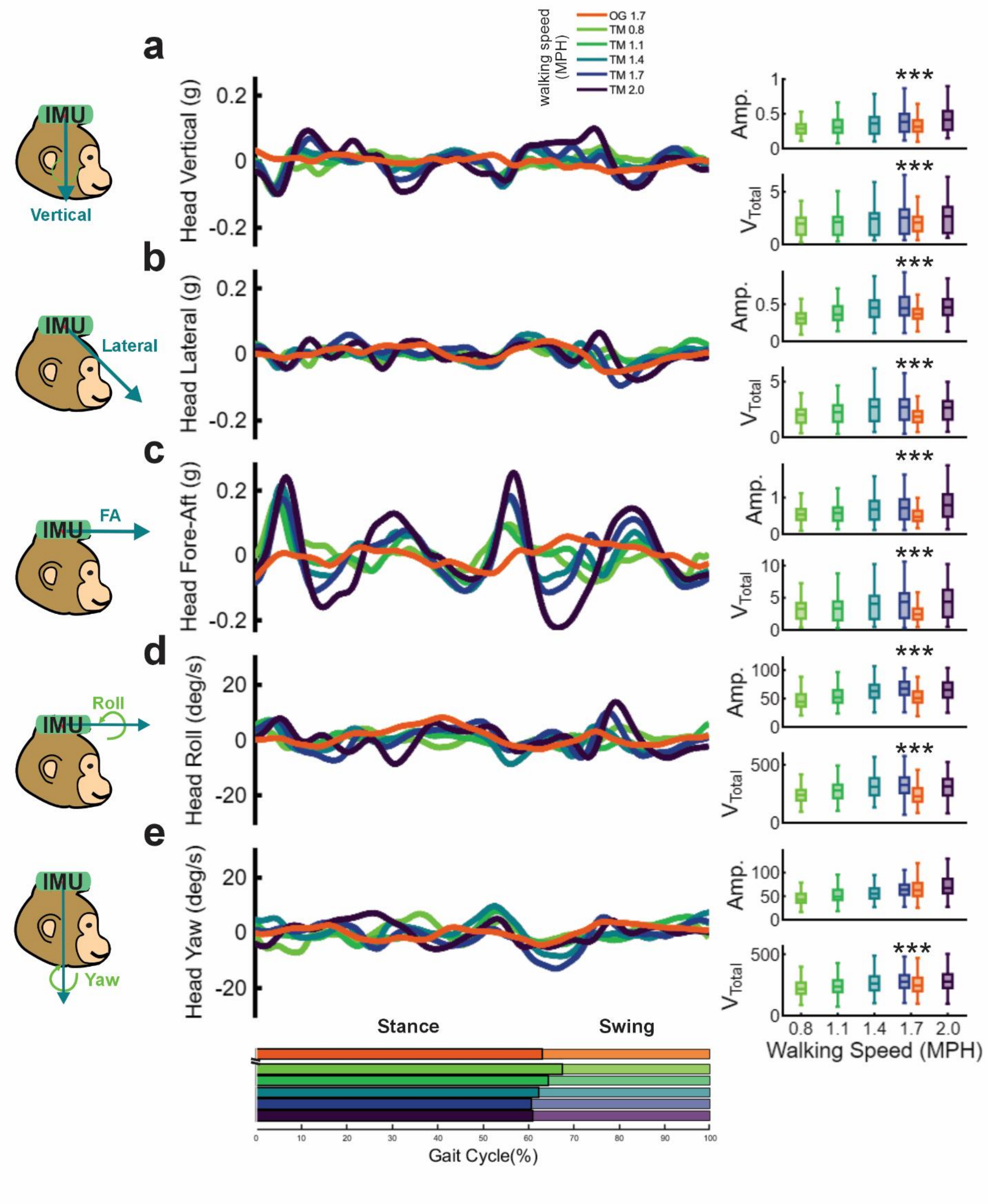


**Supplementary Figure 4:** Head rotational velocity and translational acceleration across the gait cycle. Illustrations to the left indicate the axis being plotted. Time course of averaged head vertical (a), lateral (b), fore-aft (c) acceleration, and roll (d) and yaw (e) angular velocity during the gait cycle in representative Monkey J, as in Figure 2c. Horizontal bars along the bottom indicate the

stance and swing phase for each condition. Right panels show Amp. (top) and $V_{Total}$ (bottom) for each condition from the data of all monkeys. Head motion Amp., and $V_{Total}$ increased with increasing treadmill speeds in all axes. Results are presented as boxplots, which show the median (center line), interquartile range (box), and whiskers extending to the nonoutlier (1.5 IQR) maximum and minimum. Significant differences were found between treadmill walking at matched speed and overground walking in both Amp. (all axes except yaw) and $V_{Total}$ (all axes), ***$p < 0.001$. All corresponding p values are reported in Supplementary Tables 4, 5.

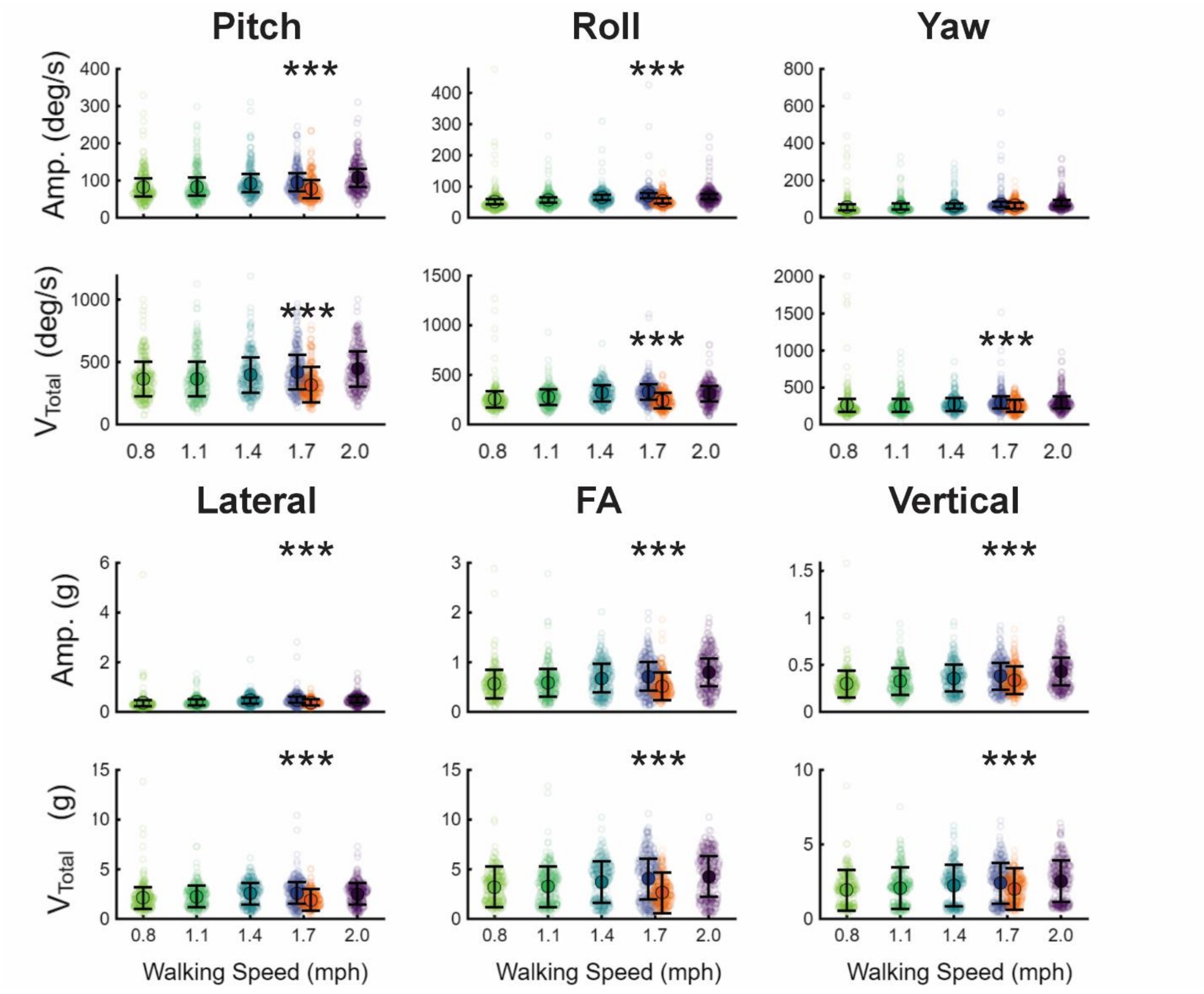


**Supplementary Figure 5:** Head-in-space rotational velocity and translational acceleration measures for all monkeys. For each axis, Amp. and $V_{Total}$ are shown as dot plots with LME-estimated means and 95% CIs. All corresponding p values are reported in Supplementary Tables 4, 5.

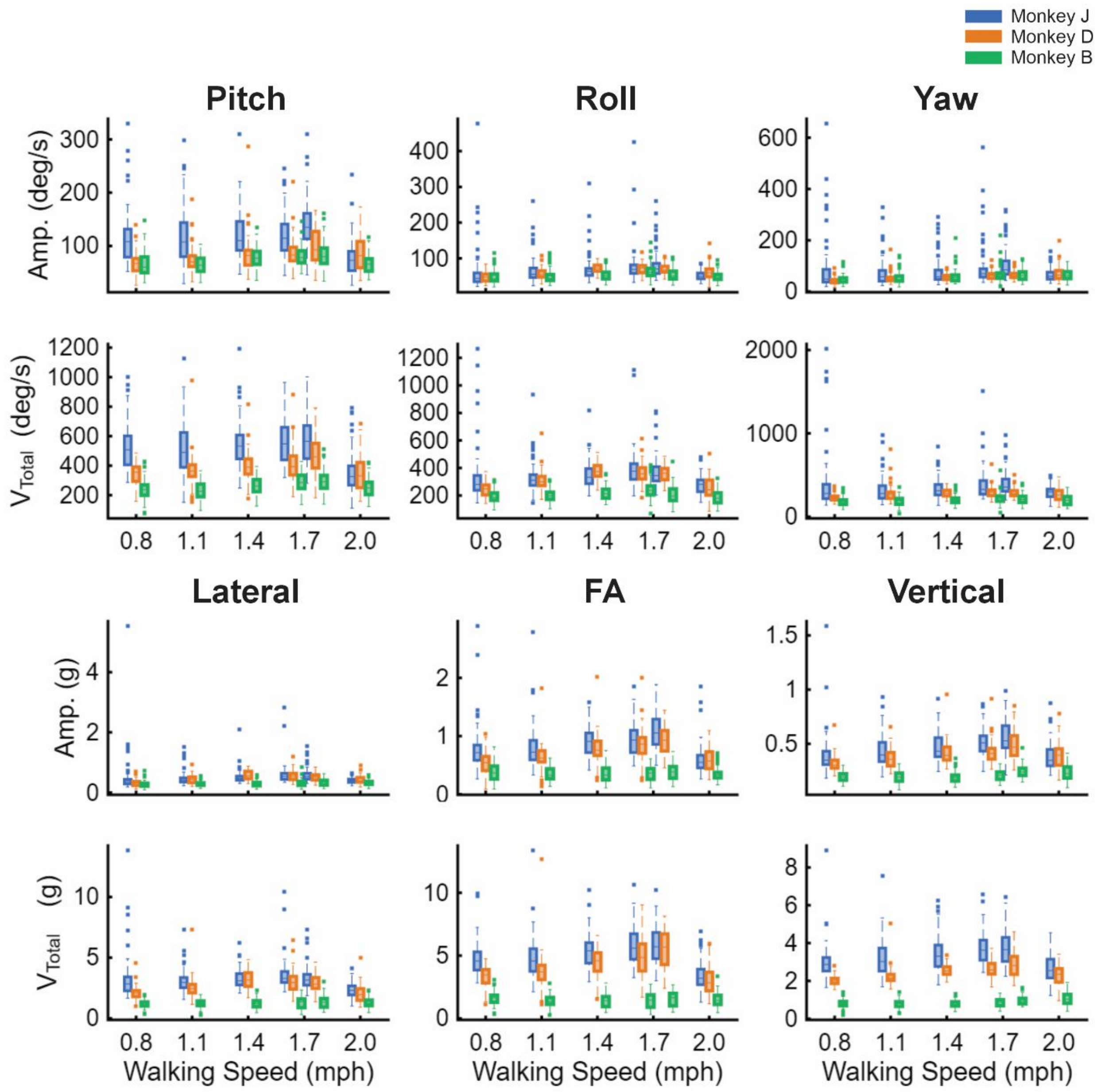


**Supplementary Figure 6:** Head-in-space rotational velocity and translational acceleration measures for each monkey. Amp. and $V_{Total}$ are shown separately for each animal in each axis (blue: Monkey J, orange: Monkey D; green: Monkey B). Monkeys exhibited similar trends across most axes. Linear regressions were applied to treadmill walking data when speed-dependent effects were significant. Results are presented as boxplots, which show the median (center line), interquartile range (box), and whiskers extending to the nonoutlier (1.5 IQR) maximum, minimum, and outliers. All corresponding r and p values are reported in Supplementary Table 6.

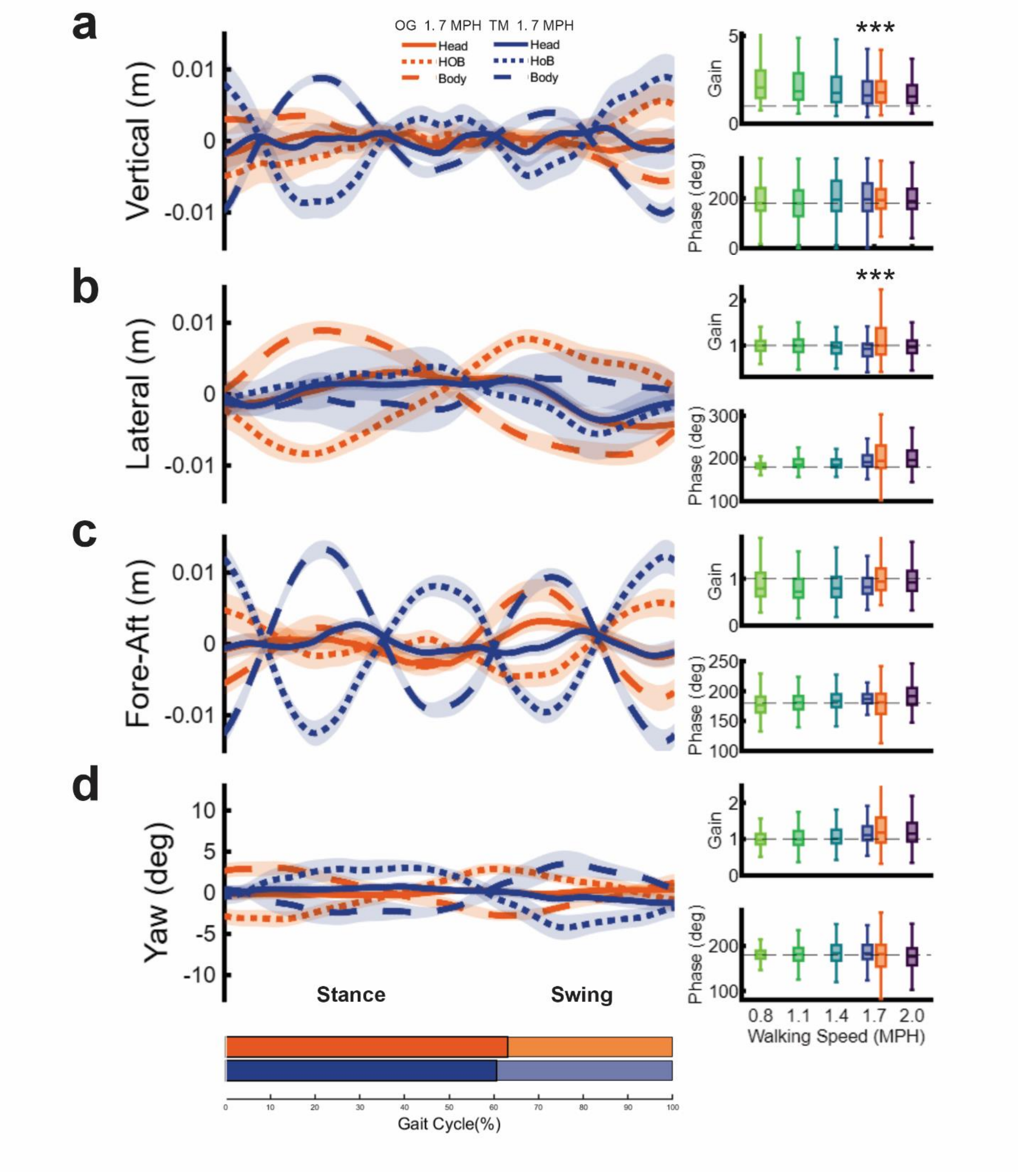


**Supplementary Figure 7:** Head-on-body movements in vertical (a), lateral (b), fore-aft (c), and yaw (d) axes across the gait cycle. Cycle-averaged motion for head-in-space (solid line), body-in-space (dashed line), and head-on-body (dotted line) are illustrated during overground walking (orange) and treadmill walking at the matched speed 1.7 MPH (blue) in representative Monkey J. Horizontal bars along the bottom indicate the stance and swing phase for these two

conditions. Right panels show the average head-on-body gain (top) and phase (bottom) for each condition from data of all monkeys. Dashed gray lines indicate perfect compensation, i.e. gain of 1 and a phase of 180 degrees. Results are presented as boxplots, which show the median (center line), interquartile range (box), and whiskers extending to the nonoutlier (1.5 IQR) maximum and minimum. All corresponding p values are reported in Supplementary Tables 7, 8 (***$p<0.001$).

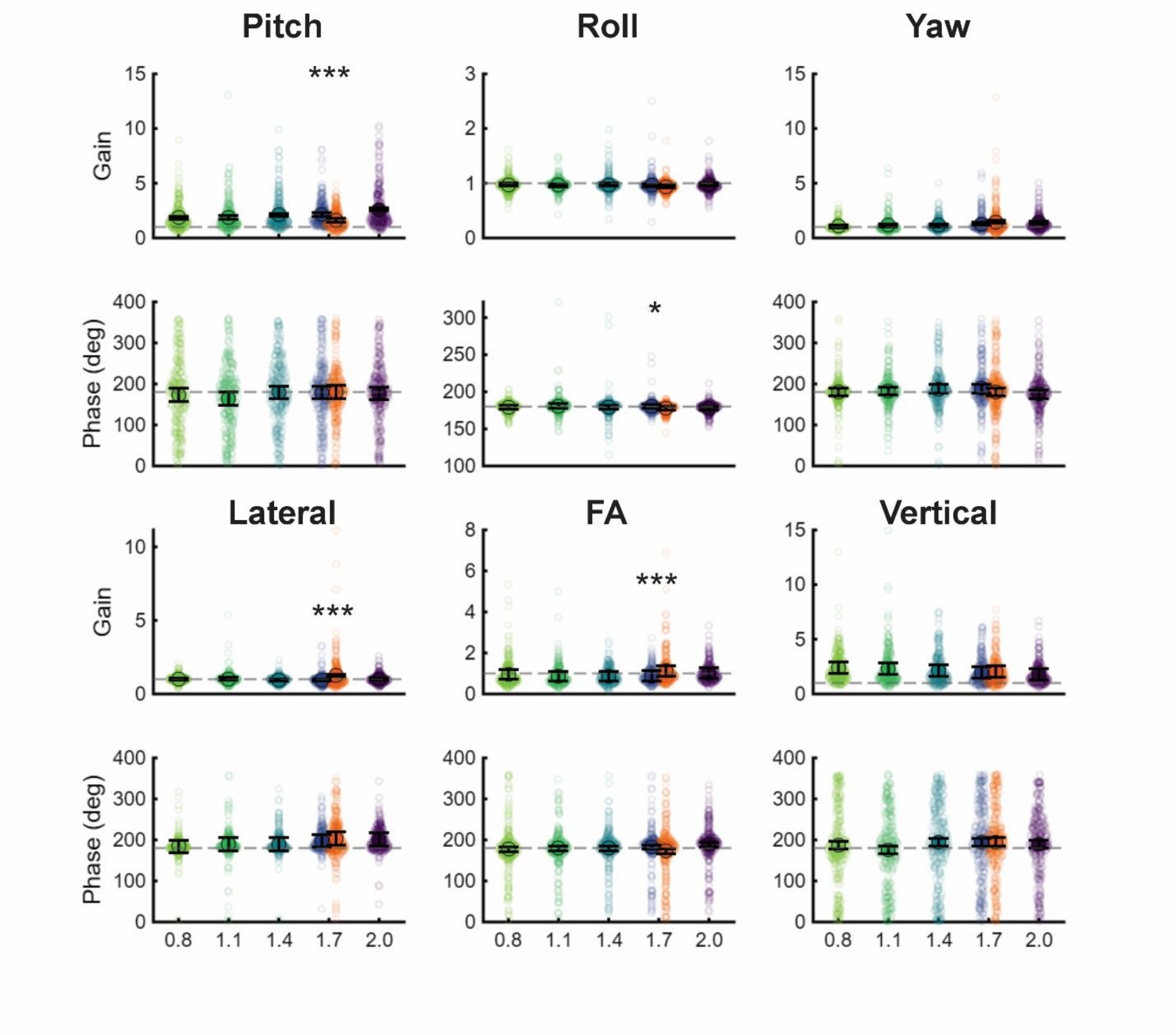


**Supplementary Figure 8:** Head-on-body gain and phase measures for all monkeys. For each axis, gain and phase are shown as dot plots with LME-estimated means and 95% CIs. All corresponding p values are reported in Supplementary Tables 7, 8.

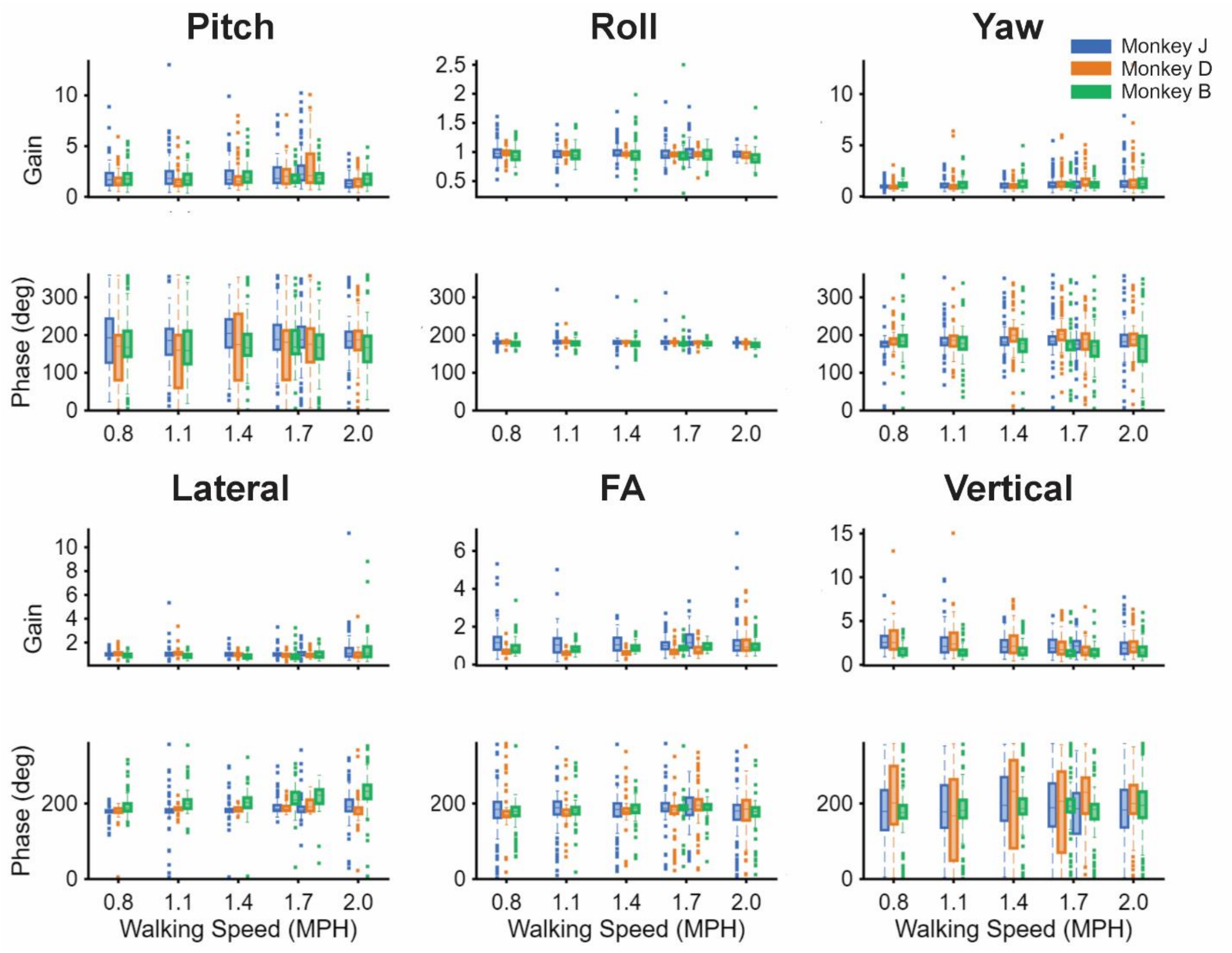


**Supplementary Figure 9:** Head-on-body gain and phase for each animal in each axis (blue: Monkey J, orange: Monkey D; green: Monkey B). Monkeys exhibited similar trends across most axes. Linear regressions were applied to treadmill walking data when speed-dependent effects were significant. Results are presented as boxplots, which show the median (center line), interquartile range (box), and whiskers extending to the nonoutlier (1.5 IQR) maximum, minimum, and outliers. All corresponding r and p values are reported in Supplementary Table 9.

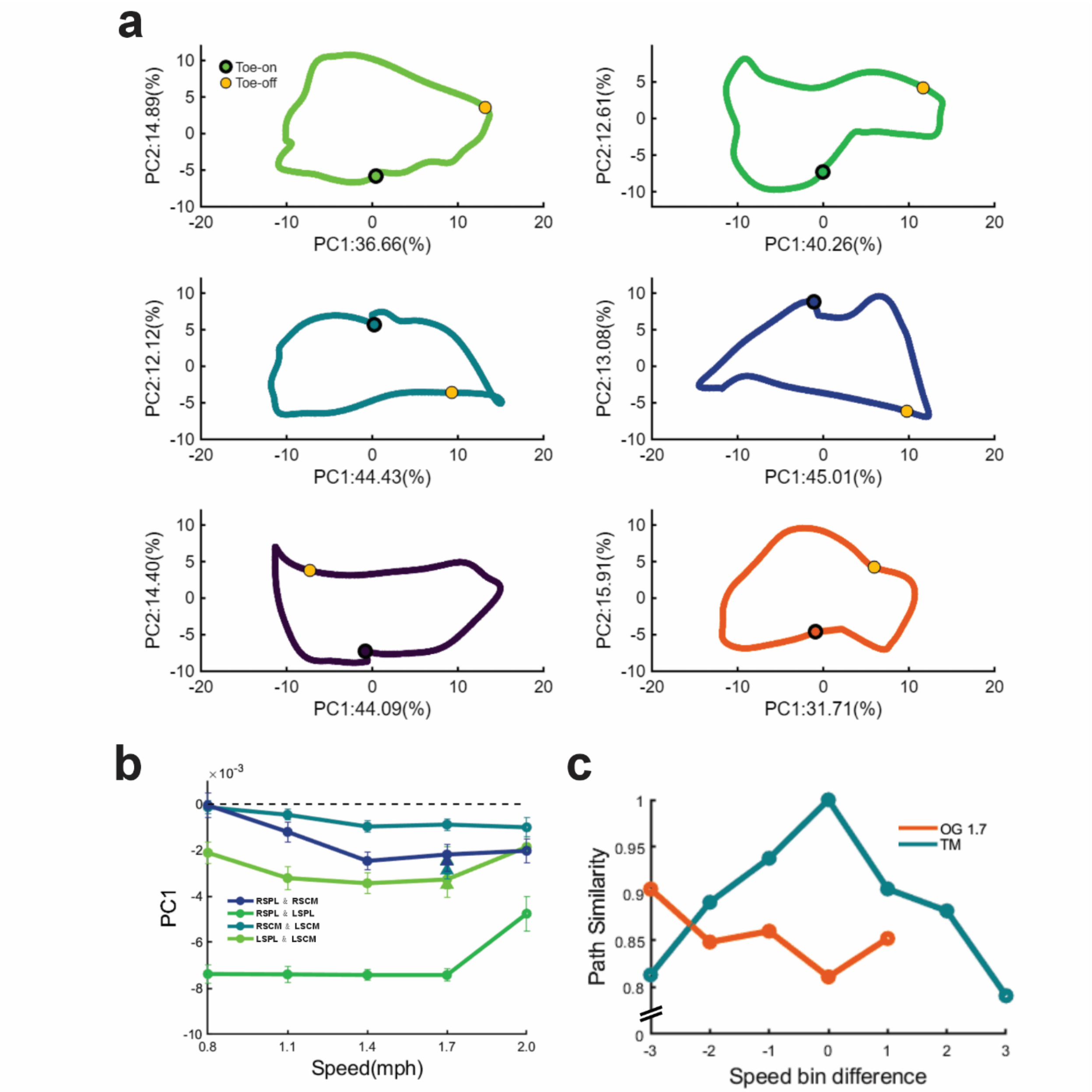


**Supplementary Figure 10:** Neck muscles demonstrated consistent population activation patterns. (a) The geometry of neck muscle population responses in PCA space shows that neck muscles exhibit similar cyclic activation patterns across conditions. Comparison of individual-condition muscle recruitment trajectories were created by projecting the population response onto the top two PCs separately within each condition. Each point within a trajectory represents a time point within the stride cycle (yellow: swing phase onset; black: swing phase end). (b) Averaged product of factors loading on PC1 for antagonistic muscle pairs (LSPL-LSCM, LSPL-RSPL, LSCM-RSCM, RSPL-RSCM) in each condition. Negative values indicate that antagonist pairs consistently have opposite contributions to the first PC. (c) Average path similarity (quantified in

the top six PCs) decreases with increasing speed difference and is generally higher within treadmill conditions than between treadmill and overground walking, even at the matched speed, except for the biggest speed difference -3 (speed 0.8 MPH).

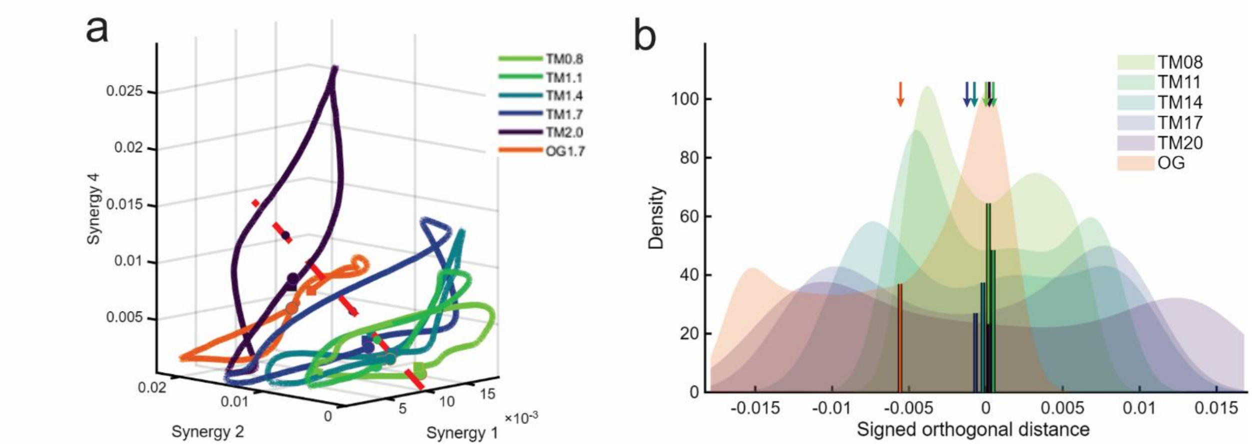


**Supplementary Figure 11:** Neck muscle population activation patterns show similar trends across analysis methods. (a) 3D plots of each condition's mean muscle activity projected into a space defined by Synergy 1, 2, and 4, extracted via NNMF. (b) Signed orthogonal distance distributions again show displacement of overground walking relative to treadmill conditions. Vertical lines mark the condition means. Distances are residuals to the reference line projected onto Synergy 4.

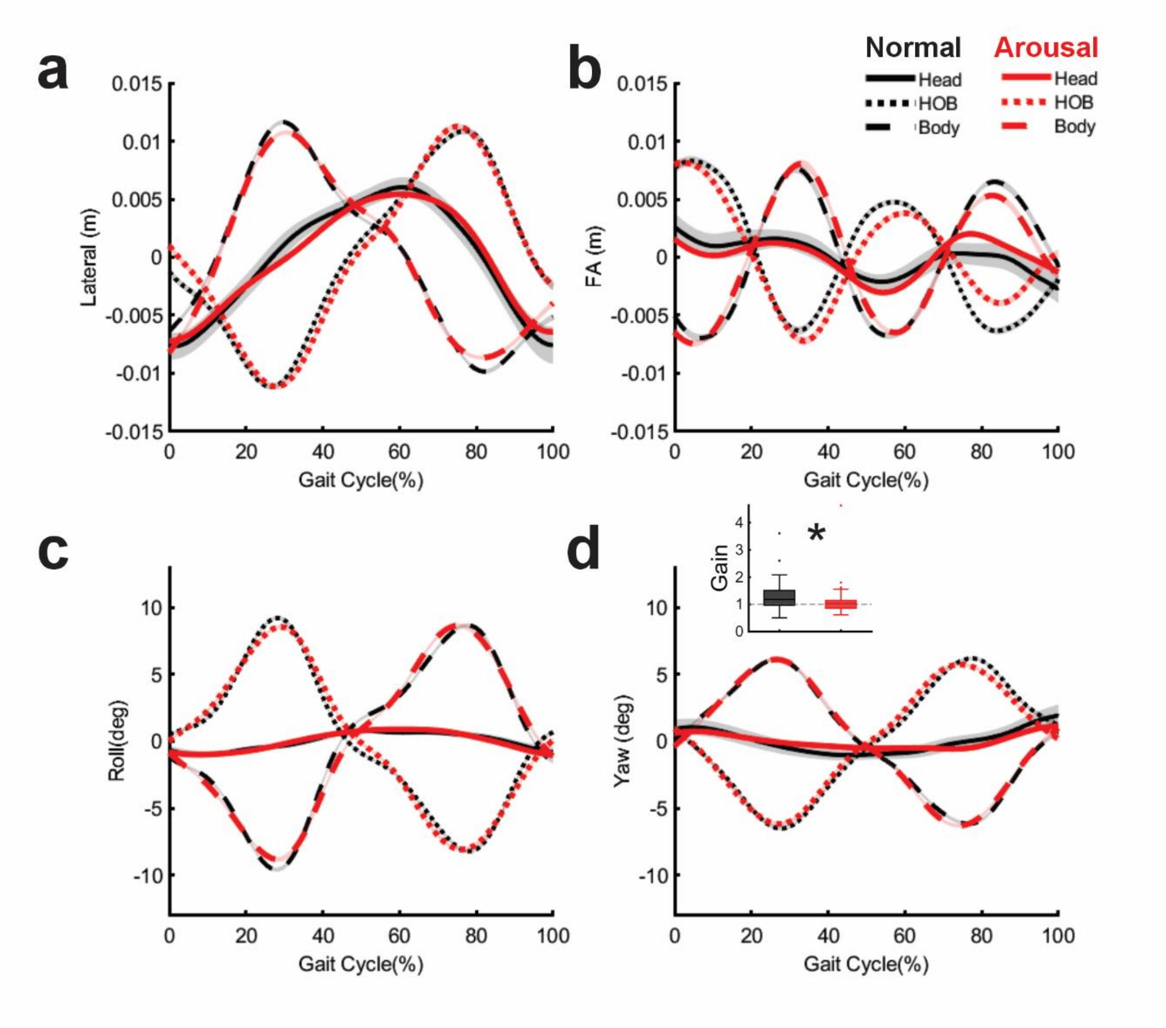


**Supplementary Figure 12:** Autonomic arousal effects on the compensatory head-on-body motion in additional axes. Comparisons of averaged head-in-space, body-in-space, and head-on-body lateral (a), fore-aft (b), roll (c), and yaw (d) over the gait cycle during normal (black) and arousal (red) overground walking in representative Monkey B. Autonomic arousal generated significantly improved compensatory head-on-body motion specifically in yaw. Inset bar plot (d) compares the gain of compensatory head-on-body motion. All corresponding p values are reported in Supplementary Table 11 (*p<0.05).

# Tables

| Amplitude | | Roll | | | | | |
|---|---|---|---|---|---|---|---|
| P values | | TM 0.8 | TM 1.1 | TM 1.4 | TM 1.7 | TM 2.0 | OG |
| Pitch | TM 0.8 | | 1 | 1 | 1 | 1 | 1 |
| | TM 1.1 | 1 | | 1 | 1 | 1 | 1 |
| | TM 1.4 | 1 | 1 | | 1 | 1 | 0.9939 |
| | TM 1.7 | 0.079 | 0.3831 | 0.3831 | | 1 | 1 |
| | TM 2.0 | 1 | 1 | 1 | 0.0156 | | 1 |
| | OG | 7.4E-05 | 0.0011 | 0.0012 | 0.395 | 5.49E-06 | |
| P values | | Lateral | | | | | |
| | | TM 0.8 | TM 1.1 | TM 1.4 | TM 1.7 | TM 2.0 | OG |
| Yaw | TM 0.8 | | 0.0358 | 0.0757 | 5.58E-04 | 5.16E-06 | 9.89E-16 |
| | TM 1.1 | 1 | | 0.7684 | 0.6979 | 0.1299 | 3.38E-07 |
| | TM 1.4 | 1 | 1 | | 0.5472 | 0.0757 | 6.60E-08 |
| | TM 1.7 | 1 | 1 | 1 | | 0.6979 | 1.28E-04 |
| | TM 2.0 | 0.3385 | 1 | 1 | 0.3277 | | 0.0071 |
| | OG | 0.3277 | 1 | 1 | 0.3114 | 1 | |
| P values | | Vertical | | | | | |
| | | TM 0.8 | TM 1.1 | TM 1.4 | TM 1.7 | TM 2.0 | OG |
| FA | TM 0.8 | | 1 | 1 | 0.3887 | 0.0145 | 5.21E-08 |
| | TM 1.1 | 1 | | 1 | 1 | 0.1875 | 7.44E-06 |
| | TM 1.4 | 1 | 1 | | 1 | 0.3887 | 3.76E-05 |
| | TM 1.7 | 0.0146 | 0.0053 | 0.1661 | | 1 | 8.13E-04 |
| | TM 2.0 | 1 | 1 | 1 | 0.101 | | 0.0659 |
| | OG | 0.4376 | 0.7303 | 0.057 | 3.59E-06 | 0.101 | |

**Supplementary Table 1:** Post hoc all-pairs comparisons of head position amplitude. For each axis, pairwise test and Holm–Bonferroni–adjusted p values are reported. Analyses are presented for all monkeys combined. For each axis, N=1800, df=1794. Related to Fig. 2 and Supplementary Figs. 1–2. Red: $p<0.001$; Light red: $p<0.01$; Light pink: $p<0.05$.

| Total Variation | | Roll | | | | | |
|---|---|---|---|---|---|---|---|
| P values | | TM 0.8 | TM 1.1 | TM 1.4 | TM 1.7 | TM 2.0 | OG |
| Pitch | TM 0.8 | | 0.951 | 0.3829 | 1 | 0.0177 | 0.0012 |
| | TM 1.1 | 0.0295 | | 1 | 1 | 0.6596 | 0.1703 |
| | TM 1.4 | 0.0022 | 1 | | 1 | 1 | 0.5504 |
| | TM 1.7 | 6.71E-07 | 0.064 | 0.384 | | 0.3552 | 0.0558 |
| | TM 2.0 | 0.0013 | 1 | 1 | 0.4255 | | 1 |
| | OG | 1.74E-32 | 1.09E-19 | 1.01E-16 | 6.73E-11 | 3.41E-16 | |
| P values | | Lateral | | | | | |
| | | TM 0.8 | TM 1.1 | TM 1.4 | TM 1.7 | TM 2.0 | OG |
| Yaw | TM 0.8 | | 5.54E-04 | 0.009 | 0.0881 | 1 | 0.0124 |
| | TM 1.1 | 1 | | 1 | 1 | 0.0974 | 1 |
| | TM 1.4 | 1 | 1 | | 1 | 0.5722 | 1 |
| | TM 1.7 | 1 | 1 | 1 | | 1 | 1 |
| | TM 2.0 | 1 | 0.8254 | 1 | 1 | | 0.6575 |
| | OG | 0.4808 | 0.0782 | 1 | 1 | 1 | |
| P values | | Vertical | | | | | |
| | | TM 0.8 | TM 1.1 | TM 1.4 | TM 1.7 | TM 2.0 | OG |
| FA | TM 0.8 | | 0.3688 | 0.0172 | 1.98E-04 | 2.14E-08 | 1.49E-13 |
| | TM 1.1 | 1 | | 0.3688 | 0.0662 | 1.70E-04 | 2.14E-08 |
| | TM 1.4 | 0.0046 | 0.0513 | | 0.3688 | 0.0195 | 2.86E-05 |
| | TM 1.7 | 5.79E-08 | 3.92E-06 | 0.0923 | | 0.3688 | 0.0039 |
| | TM 2.0 | 6.40E-09 | 6.27E-07 | 0.0417 | 1 | | 0.3688 |
| | OG | 2.64E-06 | 1.13E-04 | 0.4286 | 1 | 1 | |

**Supplementary Table 2:** Post hoc all-pairs comparisons of head position total variation. For each axis, pairwise test and Holm–Bonferroni–adjusted p values are reported. Analyses are presented for all monkeys combined. For each axis, N=1800, df=1794. Related to Fig. 2 and Supplementary Figs. 1–2. Red: p<0.001; Light red: p<0.01; Light pink: p<0.05.

| Monkey | Parameters | | Pitch | Roll | Yaw | Lateral | Fore-aft | Vertical |
|---|---|---|---|---|---|---|---|---|
| Monkey J | Amp. | r | -0.033 | -0.017 | 0.076 | 0.024 | 0.015 | -0.099 |
| | | p | 0.458 | 0.701 | 0.089 | 0.560 | 0.730 | 0.028 |
| | $V_{Total}$ | r | -0.189 | -0.126 | -0.018 | -0.041 | -0.196 | -0.188 |
| | | p | 2.0E-5 | 0.005 | 0.687 | 0.356 | 1.0E-5 | 2.2E-5 |
| Monkey D | Amp. | r | 0.048 | -0.014 | 0.008 | 0.098 | 0.054 | -0.105 |
| | | p | 0.281 | 0.760 | 0.857 | 0.029 | 0.231 | 0.018 |
| | $V_{Total}$ | r | -0.160 | -0.089 | -0.207 | -0.073 | -0.171 | -0.268 |
| | | p | 3.2E-4 | 4.7E-2 | 3.0E-6 | 0.104 | 1.3E-4 | 1.2E-9 |
| Monkey B | Amp. | r | -0.030 | 0.015 | -0.016 | 0.348 | -0.391 | -0.103 |
| | | p | 0.505 | 0.735 | 0.720 | 1.1E-15 | 1.1E-19 | 0.021 |
| | $V_{Total}$ | r | 0.056 | 0.052 | -0.044 | 0.176 | -0.502 | -0.210 |
| | | p | 0.211 | 0.246 | 0.322 | 7.6E-5 | 2.9E-33 | 2.2E-6 |
| **Monkey** | **Parameters** | | **Pitch** | **Roll** | **Yaw** | **Lateral** | **Fore-aft** | **Vertical** |
| Monkey J | Amp. | p | 1.8E-5 | 1.4E-4 | 0.923 | 0.194 | 0.795 | 6.1E-8 |
| | $V_{Total}$ | p | 2.2E-11 | 1.8E-6 | 0.001 | 0.224 | 0.027 | 4.4E-5 |
| Monkey D | Amp. | p | 1.7E-7 | 3.9E-5 | 1.6E-11 | 6.4E-8 | 5.9E-14 | 7.3E-4 |
| | $V_{Total}$ | p | 0.043 | 0.104 | 4.4E-8 | 0.791 | 7.0E-6 | 0.346 |
| Monkey B | Amp. | p | 0.122 | 9.5E-4 | 2.5E-4 | 0.005 | 0.008 | 0.004 |
| | $V_{Total}$ | p | 2.6E-9 | 0.997 | 0.941 | 0.061 | 0.431 | 0.693 |

**Supplementary Table 3:** Top rows show correlations between head position measures (Amp. and $V_{Total}$) and treadmill walking speed for each monkey. Linear regression correlation coefficients (r) and corresponding p-values are shown for each axis. For each axis, N=100, df=98.

Bottom rows show p-values for those same measures comparing overground walking versus speed-matched treadmill walking. Distributional properties were assessed using the Lilliefors test, followed by either a t-test or Wilcoxon rank-sum test as appropriate. For each axis, N=200, df=198. Red: p<0.001; Light red: p<0.01; Light pink: p<0.05.

| Amplitude | | Roll | | | | | |
|---|---|---|---|---|---|---|---|
| P values | | TM 0.8 | TM 1.1 | TM 1.4 | TM 1.7 | TM 2.0 | OG |
| | TM 0.8 | | 0.0823 | 2.28E-09 | 4.42E-17 | 4.70E-13 | 0.7471 |
| | TM 1.1 | 0.8005 | | 5.94E-04 | 3.17E-09 | 2.23E-06 | 0.7471 |
| | TM 1.4 | 2.17E-04 | 0.0026 | | 0.0823 | 0.7471 | 3.24E-06 |
| | TM 1.7 | 6.18E-06 | 1.19E-04 | 0.8005 | | 0.7471 | 1.43E-12 |
| | TM 2.0 | 6.81E-20 | 2.43E-17 | 8.00E-07 | 4.62E-05 | | 3.18E-09 |
| | OG | 0.3262 | 0.0922 | 1.18E-07 | 7.03E-10 | 1.01E-26 | |
| P values | | Lateral | | | | | |
| | | TM 0.8 | TM 1.1 | TM 1.4 | TM 1.7 | TM 2.0 | OG |
| Yaw | TM 0.8 | | 0.0993 | 2.71E-09 | 3.46E-13 | 2.76E-11 | 0.5675 |
| | TM 1.1 | 0.67352 | | 4.76E-04 | 1.32E-06 | 2.38E-05 | 1 |
| | TM 1.4 | 0.15786 | 0.55554 | | 0.7694 | 1 | 1.49E-05 |
| | TM 1.7 | 1.01E-05 | 5.61E-04 | 0.05035 | | 1 | 1.44E-08 |
| | TM 2.0 | 1.30E-10 | 3.89E-08 | 4.30E-05 | 0.27914 | | 4.71E-07 |
| | OG | 0.01328 | 0.15646 | 0.67352 | 0.30462 | 2.33E-03 | |
| P values | | Vertical | | | | | |
| | | TM 0.8 | TM 1.1 | TM 1.4 | TM 1.7 | TM 2.0 | OG |
| FA | TM 0.8 | | 0.0056 | 2.09E-10 | 7.40E-20 | 1.99E-47 | 6.42E-05 |
| | TM 1.1 | 0.2546 | | 0.002 | 3.23E-09 | 2.33E-30 | 0.224 |
| | TM 1.4 | 2.82E-08 | 1.18E-05 | | 0.0174 | 1.36E-15 | 0.04 |
| | TM 1.7 | 1.66E-13 | 4.88E-10 | 0.1358 | | 2.00E-07 | 2.77E-06 |
| | TM 2.0 | 3.44E-31 | 7.00E-26 | 7.01E-09 | 7.63E-05 | | 8.91E-25 |
| | OG | 0.0729 | 0.0028 | 6.53E-15 | 7.70E-22 | 5.31E-43 | |

**Supplementary Table 4:** Post hoc all-pairs comparisons of head rotational velocity or translational acceleration amplitude. For each axis, pairwise test and Holm–Bonferroni–adjusted p-values are reported. Analyses are presented for all monkeys combined. For each axis, N=1800, df=1794. Related to Fig. 2 and Supplementary Figs. 4–5. Red: $p<0.001$; Light red: $p<0.01$; Light pink: $p<0.05$.

| Total Variation | | Roll | | | | | |
|---|---|---|---|---|---|---|---|
| P values | | TM 0.8 | TM 1.1 | TM 1.4 | TM 1.7 | TM 2.0 | OG |
| Pitch | TM 0.8 | | 0.0071 | 1.40E-17 | 9.23E-27 | 7.83E-15 | 0.0863 |
| | TM 1.1 | 0.9501 | | 1.06E-07 | 3.12E-14 | 5.23E-06 | 1.40E-06 |
| | TM 1.4 | 0.003 | 0.0029 | | 0.0771 | 0.4391 | 7.54E-26 |
| | TM 1.7 | 9.08E-08 | 7.00E-08 | 0.0352 | | 0.0107 | 1.17E-36 |
| | TM 2.0 | 8.23E-16 | 5.32E-16 | 3.86E-06 | 0.0225 | | 1.62E-22 |
| | OG | 4.56E-06 | 5.37E-06 | 1.22E-15 | 5.12E-25 | 4.70E-38 | |
| P values | | Lateral | | | | | |
| | | TM 0.8 | TM 1.1 | TM 1.4 | TM 1.7 | TM 2.0 | OG |
| Yaw | TM 0.8 | | 0.3811 | 2.81E-10 | 2.10E-12 | 9.54E-08 | 0.0012 |
| | TM 1.1 | 1 | | 3.52E-06 | 9.54E-08 | 2.62E-04 | 7.64E-07 |
| | TM 1.4 | 0.754 | 0.6064 | | 0.6877 | 0.6877 | 1.70E-23 |
| | TM 1.7 | 5.95E-04 | 2.98E-04 | 0.0781 | | 0.3811 | 1.30E-26 |
| | TM 2.0 | 2.98E-04 | 1.35E-04 | 0.0493 | 1 | | 1.28E-19 |
| | OG | 1 | 1 | 0.2681 | 4.75E-05 | 1.97E-05 | |
| P values | | Vertical | | | | | |
| | | TM 0.8 | TM 1.1 | TM 1.4 | TM 1.7 | TM 2.0 | OG |
| FA | TM 0.8 | | 0.0122 | 3.04E-10 | 2.07E-20 | 6.67E-34 | 0.2471 |
| | TM 1.1 | 0.6278 | | 0.0016 | 4.37E-10 | 3.36E-20 | 0.2471 |
| | TM 1.4 | 6.81E-07 | 6.65E-06 | | 0.0122 | 2.73E-08 | 2.07E-06 |
| | TM 1.7 | 3.24E-17 | 1.66E-15 | 0.0014 | | 0.0122 | 1.01E-14 |
| | TM 2.0 | 1.57E-25 | 1.89E-23 | 1.87E-07 | 0.0803 | | 1.59E-26 |
| | OG | 1.56E-07 | 1.01E-08 | 9.15E-26 | 1.64E-43 | 1.10E-55 | |

**Supplementary Table 5:** Post hoc all-pairs comparisons of head rotational velocity or translational acceleration total variation. For each axis, pairwise test and Holm–Bonferroni–adjusted p-values are reported. Analyses are presented for all monkeys combined. For each axis, N=1800, df=1794. Related to Fig. 2 and Supplementary Figs. 4–5. Red: $p<0.001$; Light red: $p<0.01$; Light pink: $p<0.05$.

| Monkey | Parameters | | Pitch | Roll | Yaw | Lateral | Fore-aft | Vertical |
|---|---|---|---|---|---|---|---|---|
| | | | | | | | | |
| **Monkey J** | **Amp.** | **r** | 0.179 | 0.169 | 0.141 | 0.131 | 0.341 | 0.403 |
| | | **p** | 5.7E-5 | 1.5E-4 | 0.002 | 0.003 | 4.1E-15 | 6.7E-21 |
| | **$V_{Total}$** | **r** | 0.149 | 0.187 | 0.089 | 0.111 | 0.299 | 0.327 |
| | | **p** | 8.2E-4 | 2.7E-5 | 0.047 | 0.013 | 8.5E-12 | 6.0E-14 |
| **Monkey D** | **Amp.** | **r** | 0.394 | 0.509 | 0.480 | 0.447 | 0.521 | 0.502 |
| | | **p** | 5.7E-20 | 2.5E-34 | 3.3E-30 | 5.9E-26 | 3.9E-36 | 2.9E-33 |
| | **$V_{Total}$** | **r** | 0.398 | 0.503 | 0.301 | 0.380 | 0.523 | 0.596 |
| | | **p** | 2.1E-20 | 2.4E-33 | 6.6E-12 | 1.3E-18 | 2.1E-36 | 1.9E-49 |
| **Monkey B** | **Amp.** | **r** | 0.332 | 0.218 | 0.305 | 0.206 | 0.001 | 0.311 |
| | | **p** | 2.7E-14 | 8.2E-7 | 3.2E-12 | 3.5E-6 | 0.987 | 1.1E-12 |
| | **$V_{Total}$** | **r** | 0.353 | 0.151 | 0.259 | 0.101 | 0.004 | 0.238 |
| | | **p** | 4.3E-16 | 7.0E-4 | 4.4E-9 | 2.4E-2 | 0.937 | 7.1E-8 |
| **Monkey** | **Parameters** | | **Pitch** | **Roll** | **Yaw** | **Lateral** | **Fore-aft** | **Vertical** |
| **Monkey J** | **Amp.** | **p** | 2.0E-13 | 1.6E-13 | 0.001 | 2.0E-17 | 1.7E-19 | 6.2E-14 |
| | **$V_{Total}$** | **p** | 1.8E-20 | 3.4E-17 | 2.7E-5 | 8.1E-23 | 1.1E-23 | 3.8E-21 |
| **Monkey D** | **Amp.** | **p** | 0.772 | 2.1E-5 | 0.150 | 2.6E-9 | 1.0E-10 | 0.109 |
| | **$V_{Total}$** | **p** | 9.5E-5 | 5.4E-17 | 0.006 | 1.3E-15 | 7.5E-16 | 2.7E-5 |
| **Monkey B** | **Amp.** | **p** | 1.7E-9 | 8.4E-11 | 0.688 | 0.405 | 0.378 | 0.008 |
| | **$V_{Total}$** | **p** | 1.4E-5 | 8.1E-11 | 0.004 | 0.933 | 0.419 | 4.0E-7 |

**Supplementary Table 6:** Top rows show correlations between head rotational velocity or translational acceleration measures (Amp. and $V_{Total}$) and treadmill walking speed for each monkey. Linear regression correlation coefficients (r) and corresponding p-values are shown for each axis. For each axis, N=100, df=98.

Bottom rows show p-values for those same measures comparing overground walking to speed-matched treadmill walking. Distributional properties that were assessed using the Lilliefors test, followed by either a t-test or Wilcoxon rank-sum test as appropriate. For each axis, N=200, df=198. Related to Supplementary Figure 6. Red: $p<0.001$; Light red: $p<0.01$; Light pink: $p<0.05$.

| Gain | | Roll | | | | | |
|---|---|---|---|---|---|---|---|
| P values | | TM 0.8 | TM 1.1 | TM 1.4 | TM 1.7 | TM 2.0 | OG |
| Pitch | TM 0.8 | | 1 | 1 | 1 | 1 | 0.0122 |
| | TM 1.1 | 1 | | 1 | 1 | 1 | 0.0202 |
| | TM 1.4 | 0.0726 | 0.0738 | | 1 | 1 | 0.0018 |
| | TM 1.7 | 0.0456 | 0.0726 | 1 | | 1 | 0.1021 |
| | TM 2.0 | 2.85E-12 | 1.14E-11 | 1.02E-05 | 3.44E-05 | | 0.0133 |
| | OG | 0.0738 | 0.0726 | 1.42E-05 | 3.92E-06 | 8.57E-21 | |
| P values | | Lateral | | | | | |
| | | TM 0.8 | TM 1.1 | TM 1.4 | TM 1.7 | TM 2.0 | OG |
| Yaw | TM 0.8 | | 1 | 0.9728 | 0.6992 | 1 | 2.60E-09 |
| | TM 1.1 | 1 | | 0.4544 | 0.234 | 1 | 7.20E-08 |
| | TM 1.4 | 1 | 1 | | 1 | 0.9728 | 1.61E-13 |
| | TM 1.7 | 0.0435 | 0.5727 | 0.5727 | | 0.6992 | 1.52E-14 |
| | TM 2.0 | 5.67E-04 | 0.0269 | 0.0269 | 0.98 | | 2.60E-09 |
| | OG | 4.14E-05 | 0.0039 | 0.0039 | 0.48 | 1 | |
| P values | | Vertical | | | | | |
| | | TM 0.8 | TM 1.1 | TM 1.4 | TM 1.7 | TM 2.0 | OG |
| FA | TM 0.8 | | 0.8119 | 0.1287 | 5.22E-05 | 1.83E-08 | 0.0017 |
| | TM 1.1 | 0.0399 | | 0.4398 | 0.0011 | 1.02E-06 | 0.0182 |
| | TM 1.4 | 0.0412 | 1 | | 0.161 | 0.0022 | 0.5514 |
| | TM 1.7 | 0.1182 | 1 | 1 | | 0.5514 | 0.8119 |
| | TM 2.0 | 0.65 | 3.59E-04 | 4.25E-04 | 0.0023 | | 0.1448 |
| | OG | 2.22E-04 | 3.51E-11 | 5.03E-11 | 9.18E-10 | 0.0305 | |

**Supplementary Table 7:** Post hoc all-pairs comparisons of head-on-body gain. For each axis, pairwise test and Holm–Bonferroni–adjusted p values are reported. Analyses are presented for all monkeys combined. For each axis, N=1800, df=1794. Related to Fig. 3 and Supplementary Figs. 7–8. Red: p<0.001; Light red: p<0.01; Light pink: p<0.05.

| Phase | | Roll | | | | | |
|---|---|---|---|---|---|---|---|
| P values | | TM 0.8 | TM 1.1 | TM 1.4 | TM 1.7 | TM 2.0 | OG |
| Pitch | TM 0.8 | | 0.1075 | 1 | 0.8121 | 1 | 0.4912 |
| | TM 1.1 | 1 | | 0.4053 | 1 | 0.003 | 1.30E-04 |
| | TM 1.4 | 1 | 0.2013 | | 1 | 0.6166 | 0.1415 |
| | TM 1.7 | 1 | 0.2232 | 1 | | 0.1075 | 0.0101 |
| | TM 2.0 | 1 | 0.4909 | 1 | 1 | | 1 |
| | OG | 1 | 0.1658 | 1 | 1 | 1 | |
| P values | | Lateral | | | | | |
| | | TM 0.8 | TM 1.1 | TM 1.4 | TM 1.7 | TM 2.0 | OG |
| Yaw | TM 0.8 | | 0.1743 | 0.0496 | 3.76E-07 | 1.73E-10 | 3.22E-13 |
| | TM 1.1 | 1 | | 0.7532 | 0.0033 | 1.71E-05 | 1.97E-07 |
| | TM 1.4 | 0.4753 | 0.9361 | | 0.0252 | 2.79E-04 | 5.05E-06 |
| | TM 1.7 | 0.4533 | 0.9361 | 1 | | 0.6209 | 0.1597 |
| | TM 2.0 | 0.9361 | 0.3674 | 0.0059 | 0.0044 | | 0.7532 |
| | OG | 1 | 1 | 0.4753 | 0.4533 | 0.9361 | |
| P values | | Vertical | | | | | |
| | | TM 0.8 | TM 1.1 | TM 1.4 | TM 1.7 | TM 2.0 | OG |
| FA | TM 0.8 | | 1 | 1 | 1 | 1 | 1 |
| | TM 1.1 | 1 | | 0.1303 | 0.1293 | 0.5781 | 0.1063 |
| | TM 1.4 | 1 | 1 | | 1 | 1 | 1 |
| | TM 1.7 | 1 | 1 | 1 | | 1 | 1 |
| | TM 2.0 | 0.05257 | 0.19243 | 0.29779 | 0.96373 | | 1 |
| | OG | 1 | 0.77841 | 0.55877 | 0.1325 | 5.80E-04 | |

**Supplementary Table 8:** Post hoc all-pairs comparisons of the head-on-body phase. For each axis, pairwise test and Holm–Bonferroni–adjusted p values are reported. Analyses are presented for all monkeys combined. For each axis, N=1800, df=1794. Related to Fig. 3 and Supplementary Figs. 7–8. Red: $p<0.001$; Light red: $p<0.01$; Light pink: $p<0.05$.

| Monkey | Parameter | | Pitch | Roll | Yaw | Lateral | Fore-aft | Vertical |
|---|---|---|---|---|---|---|---|---|
| Monkey J | Gain | r | 0.165 | 0.005 | 0.168 | 0.006 | -0.028 | -0.151 |
| | | p | 2.2E-4 | 0.907 | 1.6E-4 | 0.886 | 0.533 | 6.8E-4 |
| | Phase | r | 0.025 | -0.050 | 0.007 | 0.168 | 0.068 | 0.021 |
| | | p | 0.571 | 0.262 | 0.871 | 1.61E-4 | 0.131 | 0.634 |
| Monkey D | Gain | r | 0.320 | -0.094 | 0.159 | -0.261 | 0.223 | -0.296 |
| | | p | 2.1E-13 | 0.037 | 3.5E-4 | 3.2E-9 | 4.5E-7 | 1.4E-11 |
| | Phase | r | 0.097 | -0.137 | 0.023 | 0.319 | 0.045 | 0.071 |
| | | p | 0.030 | 0.002 | 0.602 | 2.8E-13 | 0.311 | 0.111 |
| Monkey B | Gain | r | 0.028 | 0.015 | 0.025 | 0.068 | 0.090 | -0.031 |
| | | p | 0.531 | 0.743 | 0.577 | 0.130 | 0.044 | 0.485 |
| | Phase | r | -0.023 | 0.019 | -0.121 | 0.257 | 0.162 | 0.031 |
| | | p | 0.615 | 0.664 | 0.007 | 5.5E-9 | 2.7E-4 | 0.494 |
| **Monkey** | **Parameters** | | **Pitch** | **Roll** | **Yaw** | **Lateral** | **Fore-aft** | **Vertical** |
| Monkey J | Gain | p | 2.3E-10 | 0.739 | 0.191 | 0.029 | 0.472 | 0.263 |
| | Phase | p | 0.888 | 0.524 | 0.780 | 0.159 | 0.004 | 0.588 |
| Monkey D | Gain | p | 4.3E-6 | 0.032 | 0.884 | 0.617 | 1.25E-9 | 0.197 |
| | Phase | p | 0.198 | 5.0E-6 | 0.004 | 2.9E-4 | 0.554 | 0.821 |
| Monkey B | Gain | p | 0.475 | 5.4E-4 | 0.277 | 9.3E-8 | 0.014 | 0.096 |
| | Phase | p | 0.120 | 0.004 | 0.093 | 1.0E-5 | 4.9E-6 | 0.948 |

**Supplementary Table 9:** Top rows show the correlations between head-on-body measures (gain and phase) and treadmill walking speed for each monkey. Linear regression correlation coefficients (r) and corresponding p-values are shown for each head position axis. For each axis, N=100, df=98.

Bottom rows show p-values for those same measures comparing overground walking to speed-matched treadmill walking. Distributional properties that were assessed using the Lilliefors test, followed by either a t-test or Wilcoxon rank-sum test as appropriate. For each axis, N=200, df=198. Related to Supplementary Figure 9. Red: $p<0.001$; Light red: $p<0.01$; Light pink: $p<0.05$.

| Speed (MPH) | 0.8 | 1.1 | 1.4 | 1.7 | 2.0 |
|---|---|---|---|---|---|
| **RSPL** | 3.1E-10 | 2.4E-06 | 4E-09 | 0.008 | 0.004 |
| **LSPL** | 1.2E-12 | 8.4E-12 | 2.1E-10 | 3.3E-4 | 0.039 |
| **RSCM** | 8.3E-4 | 1.5E-05 | 0.002 | 0.870 | 0.449 |
| **LSCM** | 4.6E-08 | 2.94E-05 | 0.296 | 0.030 | 0.078 |

**Supplementary Table 10:** p-values for each neck muscle's mean motor unit firing rate during overground walking versus each treadmill speed. Distributional properties were assessed using the Lilliefors test, followed by either a t-test or Wilcoxon rank-sum test as appropriate. Related to Figure 4. Red: $p<0.001$; Light red: $p<0.01$; Light pink: $p<0.05$.

| | Lateral | Fore-aft | Vertical | Pitch | Roll | Yaw |
|---|---|---|---|---|---|---|
| **Amp.** | 0.043 | 0.083 | 0.048 | 0.055 | 0.830 | 7.93E-5 |
| **$V_{Total}$** | 0.023 | 0.049 | 0.004 | 0.056 | 0.504 | 0.0014 |

**Supplementary Table 11:** The p values for the head-in-space comparison between normal and arousal conditions during overground walking. Significant effects ($p < 0.05$) are highlighted in red. Distributional properties were assessed using the Lilliefors test, followed by either a t-test or Wilcoxon rank-sum test as appropriate. Related to Figure 6 and Supplementary Figure 12. Red: $p<0.001$; Light red: $p<0.01$; Light pink: $p<0.05$.
