# Supplementary material for: Locomotion engages context-dependent motor strategies for head stabilization in primates
Source: Commun Biol. 2026 Jan 12;9:234. doi: 10.1038/s42003-026-09512-2 (PMC12901029; doi:10.1038/s42003-026-09512-2)
Supplement: Supplementary file 2 — Description of Additional Supplementary Files [file 42003_2026_9512_MOESM2_ESM.pdf]

Description of Additional Supplementary files

File name: Supplementary Data 1

Description: Raw data for all monkeys were provided in Supplementary Data 1.
